# Mapping and analysis of laws influencing built environments for walking and cycling in Australia

**DOI:** 10.1186/s12889-022-14897-w

**Published:** 2023-01-16

**Authors:** Tracy Nau, Sean Perry, Billie Giles-Corti, William Bellew, Adrian Bauman, Ben J. Smith

**Affiliations:** 1 Prevention Research Collaboration, Charles Perkins Centre, School of Public Health, Faculty of Medicine and Health, The University of Sydney, Sydney, NSW Australia; 2 The Australian Prevention Partnership Centre, Sydney, NSW Australia; 3 The University of Sydney Law School, Sydney, NSW Australia; 4 Healthy Liveable Cities Lab, RMIT University, Centre for Urban Research, Melbourne, VIC Australia

**Keywords:** Legal epidemiology, Environment and public health, Built environment, Walking, Bicycling, Legislation, Government regulation, Public policy

## Abstract

**Background:**

Physical inactivity is a significant public health concern, with limited signs of improvement despite a global commitment to achieving the World Health Organization’s target of 15% reduction by 2030. A systems approach is required to tackle this issue, involving the creation of environments that are conducive to physical activity. Laws represent an important tool for regulating the built environment for physical activity, are a mechanism for systems change, and have the capacity to reorient the goals and rules of a system. However, they are understudied and potentially underutilised for physical activity. Scientific legal mapping is a first step towards understanding how laws could impact the built environment to facilitate greater population physical activity.

**Method:**

We conducted a legal assessment of state and territory laws in Australia, to systematically characterise how they address built environment considerations with specific relevance to walking and cycling. An interdisciplinary team of researchers with public health, law and urban planning expertise was formed to complete the multistage process. Key steps included a systematic search of laws using a combination of original legal research, consultation of secondary sources, and review and verification by an urban planning expert; development of a coding scheme; and completion of coding and quality control procedures.

**Results:**

Most jurisdictions in Australia do not currently embed objectives in primary legislation that would promote physical activity and support an integrated approach to land use and transport planning that encourages active and sustainable lifestyles. Only two jurisdictions addressed the large majority of evidence-based standards that promote active living. Of the standards addressed in law, few fully met evidence-based recommendations. While most jurisdictions legislated responsibility for enforcement of planning law, few legislated obligations for monitoring implementation.

**Conclusion:**

Increasing physical activity is a systems issue, requiring actions across multiple sectors. An in-depth examination of the legal environment is an important step towards understanding and influencing the existing physical activity system, why it may not be generating desired outcomes, and potential opportunities for improvement. Our findings reveal opportunities where laws could be strengthened to promote more active environments. Updating this dataset periodically will generate longitudinal data that could be used to evaluate the impact of these laws on the built environment and physical activity behaviours.

**Supplementary Information:**

The online version contains supplementary material available at 10.1186/s12889-022-14897-w.

## Introduction

The preamble to the constitution of the World Health Organization (WHO) stipulates that governments of Member States have a responsibility for the health of their peoples, which can only be fulfilled by the provision of adequate health and social measures [1]. Governments may use law and other policy as the strategic and implementing tools for providing the conditions that people need to be healthy [2, 3]. Policy makers may be more inclined to use law when addressing long term goals, when voluntary regimes fail to achieve strategic objectives, and when effective implementation requires the weight of enforcement that comes with law [2]. While some governments have been reluctant to use the law for public health issues such as obesity [4], others are increasingly turning to law as a necessary and powerful tool for making progress on these issues and creating a level playing field for reform [5–7]. Such an approach aligns with recommendations from the World Health Organization (WHO) Independent High-level Commission on Noncommunicable Diseases, that governments employ their full legal powers to achieve public health goals and protect their populations [8]. Perhaps the most salient example of the use of laws in support of health is in the WHO Framework Convention on Tobacco Control (WHOFCTC) – a legally binding treaty [9]. Despite calls for a global framework for public health [10], such a framework has not been established, nor is there a framework convention for physical activity (PA). However, this does not prevent us from analysing the potential role of legal approaches to advance public health through increased PA in populations.

Physical inactivity is an important public health concern that is a significant contributor towards the global burden of noncommunicable disease [11]. Minimal signs of improvement in the prevalence of population PA have been observed for decades [12, 13]. The WHO has a recommended target of a 15% reduction in physical inactivity by 2030 [14], which was adopted by Members States of the WHO [14]. The WHO Global Action Plan on Physical Activity (GAPPA) provides an evidence and practice-based framework for countries to achieve this, using a systems-based approach to address the social and environmental determinants of physical inactivity [15].

In 2021, the WHO released an advocacy brief titled ‘Fair Play’*,* identifying three interconnected barriers that are limiting progress on an effective, efficient, and sustainable PA system at scale. These were insufficient, unequal, and ineffective investment; inadequate and misaligned policy, laws, regulatory frameworks and standards; and uneven and fragmented partnerships and program delivery [16]. Its recommendations included prioritising the development of coherent policy, laws, regulatory frameworks, and standards that reduce barriers to PA and encourage people to be more active [16]. This stipulated that mandates should be put in place to bring about improvements to environments; however, there has been limited monitoring of whether this is taking place.

Law has some important advantages compared with other policy tools. For example, laws (particularly legislation enacted through a parliamentary process) are generally more enduring than other policy tools as they are more difficult to be dismantled at the whim of a change in government [2]. Policy that is embedded in law, conveys the importance of that policy and the seriousness with which the government intends to deliver it, which helps to encourage compliance and realign industry behaviours with the public’s interest [2]. Laws also carry the weight of enforcement as there are court processes through which regulated subjects can be held to account [2], although the costs of legal action and limits on standing (which generally prevents third parties from initiating proceedings), may act as practical constraints on the enforcement of laws.

Previously, we published a framework that aligns legal strategies with the WHO GAPPA policy objectives – the Regulatory Approaches to Movement, Physical Activity, Recreation, Transport and Sport (RAMPARTS) [17]. The RAMPARTS framework aims to focus researcher and policymaker attention on the broad range of legal strategies that could potentially address the WHO GAPPA objectives, not least of which is strategic objective 2 to ‘create active environments’. Legal strategies to address this objective could include: setting quality standards for the provision of footpath infrastructure; establishing funding mechanisms to support the creation and maintenance of public open spaces; setting a mandate or authority that requires decision makers to exercise their functions with regard to health (or complementary goals such as climate change and liveability); and developing administrative or procedural mechanisms that enable greater cross-agency or government coordination to achieve integrated land use and transport planning.

Surveys of built environment practitioners working in the UK and Australia have shown their support for stronger statutory mechanisms as well as detailed requirements and standards. These are expected to reduce ambiguity and scope for sidestepping recommendations, and improve the delivery of health-promoting places [18, 19]. In the Australian context, there is evidence of public support for such interventions, as revealed by recent population surveys which showed that citizens highly value supportive environments for PA [20] and believe there should be greater prioritisation of wellbeing in policy making and investment [21]. However, there is limited research describing the specific features of laws that regulate the built environment and how they may support or hinder PA. To improve such understanding within the Australian context, we conducted an audit to identify how laws in each state and territory address built environment design considerations with a specific focus on the walkability of environments [22]. Our aim in that study was to describe the legal framework that may influence the walkability of built environments in Australia, in order to understand whether walkability considerations are addressed in law at a state and territory level, the main types of legal instruments used, and the type of approaches used by the different jurisdictions to address those considerations (e.g. through setting legal objectives, principles and standards). Our audit revealed considerable variability in the coverage, level of detail, and approaches used, raising uncertainty about the scope and strength of legal support for creating walkable environments at the national level. We therefore recommended the use of scientific legal mapping as a more systematic and rigorous method for identifying relevant laws and analysing the features and variations of these laws.

Scientific legal mapping can be conducted at a single point in time (‘legal assessment’) or longitudinally (‘policy surveillance’) [23]. Potential benefits include facilitating the diffusion of legal innovations across jurisdictions, improving the navigability of laws for non-legal professionals, identifying potential policy targets for improvement, and generating new data that can in turn inform future policy making [24]. Policy surveillance is one of the key methods employed in legal epidemiology, which has been defined as the ‘study and deployment of law as a factor in the cause, distribution, and prevention of disease and injury in a population’ [25]. Because of its scientific approach, policy surveillance creates usable data that enables the impact of law to be empirically evaluated [25]. In other public health domains such as alcohol and tobacco control, there is a long history of scientific evaluation of laws, which has led to widespread implementation of evidence-based public health law interventions [25].

Prior analyses of laws impacting PA have mainly occurred in the United States in relation to environmental trail legislation [26], Complete Streets statutes [27] and comprehensive planning state statutes for rural communities [28]. To our knowledge, scientific legal mapping has not been used in Australia or to analyse, in a comprehensive and integrated way, the laws regulating the built environment for PA. This study aimed to employ scientific legal mapping to characterise the extent to which primary legislation (specifically for planning, transport, climate change and public health) addresses objectives within an integrated approach to delivering PA-promoting places, and to examine whether planning law embraces measurable standards that align with criteria known to promote active living. We chose to limit our focus to criteria that promote walking and cycling specifically, as these are known to be policy priorities in multiple jurisdictions and can be readily adopted across the lifespan and socioeconomic contexts, thereby making important contributions towards physical activity at the population level [29, 30]. We also sought to understand how planning laws in Australia address implementation and monitoring because inadequate enforcement or monitoring (including as a result of ambiguity about responsibility for these functions) can undermine the effectiveness of otherwise well-written laws [23]. Previous research has highlighted the need for monitoring of implementation, as policy ‘leakages’ have been shown to occur at various stages [31] leading to disparities in urban planning outcomes. For example, it has been found in one Australian jurisdiction, that higher levels of implementation and compliance with a policy to enhance neighbourhood liveability was associated with better on-the-ground urban design and walking outcomes [31].

Our aim in this study was to establish a baseline assessment of how laws currently address walking and cycling, and how they compare across Australian states and territories. Detailed coding of attributes in laws makes it easier to identify differences across jurisdictions. The systematic methods stated in our protocol (see Supplementary Material) will also enable replication at future timepoints, which will allow changes in law to be tracked over time, and generate longitudinal data that can be used in the future evaluation of these laws, including their impact upon built environment and PA outcomes.

## Methods

A detailed description of the methods used is set out in our protocol (see Supplementary Material). A summary is provided below.

### Scope

A legal assessment was conducted of laws addressing built environment considerations in each Australian state and territory that are relevant to walking and cycling, in effect as of 29 November 2021. ‘Laws’ were defined as any policy with legal enforceability (e.g. Acts, Regulations and statutory instruments being those documents attaining legal enforceability under an enabling Act, Regulation or other legally binding instrument). The assessment excluded non-statutory policies (e.g. guidelines and operational policies) as these do not have legal enforceability, although non-statutory policies that were given legal weight under legislation or other legally binding instrument were eligible for inclusion. We included laws with state- or territory-wide application and region-level instruments covering the capital city and greater metropolitan. Local-level laws or laws applying to small-scale geographical areas (e.g., municipalities) were excluded. The assessment also excluded any draft instruments or instruments enacted after 29 November 2021.

### Identification of relevant laws

To identify relevant laws, we started with the results of our initial study completed in April 2021 [22] which identified the key legislative and statutory instruments in each state and territory that addressed built environment design considerations for walking. These instruments were identified by reviewing the ‘statutory policies’ and ‘legislation/regulation’ listed on the National Heart Foundation’s Healthy Active by Design website [32] and reviewing government planning websites for potentially relevant documents [22]. This was supplemented with a review of the Planning Acts in each state and territory to identify other statutory instruments enabled by the Act, which were then retrieved from government planning websites to review. The list of legislative and statutory policies included by the Centre for Urban Research in its ‘Creating Liveable Cities’ (2017) report were also reviewed [33]. The proposed list of laws was reviewed by co-author BGC, a subject matter expert in healthy liveable planning, and no further documents were identified. Any additional laws subsequently identified during coding by TN or SP were included after discussing and reviewing their relevance, and with mutual agreement. A complete list of the laws included in this study, is set out in Appendix 1 of our protocol (see Supplementary Material).

### Development of the coding scheme

The areas of focus for the legal assessment were conceptualised in consultation with BGC and guided by the issues identified in recent peer-reviewed and grey literature as issues of legal relevance for delivering PA-promoting built environment outcomes in Australia [18, 31]. These covered: 1) the extent of legislative support for an integrated approach to delivering PA-promoting outcomes; 2) the extent to which state and territory laws contained defined and measurable standards for promoting active communities through walking and cycling; 3) the alignment of standards with evidence-based recommendations (as set out in the Healthy Liveable Cities Lab’s Urban Liveability Checklist [34] and the Institute for Transportation & Development Policy (ITDP) Pedestrians First Guide) [35]; and 4) how planning legislation address implementation and monitoring. TN conducted background research to investigate these areas for each jurisdiction. This informed the development of the coding scheme, which was finalised through discussion with BGC and created in the MonQcle web-based software coding platform for legal epidemiology [36].

### Coding methods and quality control

TN and SP conducted the coding independently for all variables and all eight state territory jurisdictions in MonQcle, blinded to each other’s results. The coding scheme was refined by the coders (TN, SP) during coding, to accommodate newly identified features in the data, and completed jurisdictions were recoded accordingly. Each jurisdiction was coded using a set of coding conventions which are available in Appendix 2 of our protocol (see Supplementary Material). Caution notes were recorded in MonQcle where an explanation of the legal text was required (e.g. where the law was unclear, or the response was subject to qualification or to proposed change under a draft law). To be considered a ‘standard’, provisions needed to contain sufficient specificity to be objectively measurable (e.g. by incorporating numerical measures such as specifying the desirable distance of dwellings from an activity centre (i.e. community hubs for services, employment, transport and social interaction) [37], or the number of bicycle parking spaces to be provided in new residential apartments).

Data were periodically exported into a Microsoft Excel spreadsheet, and reviewed by TN for completeness of responses and to calculate the divergence rate. After coding one jurisdiction, the rate of divergence was 5.5%. Following clarifications to the coding scheme and conventions and coding a further two jurisdictions, the divergence rate was 2.3%. The remaining five jurisdictions were then coded, with a divergence rate of 11.2%. All divergences were resolved through discussion between TN and SP. Whenever the coding scheme or conventions were refined, TN and SP revisited the dataset to ensure coding was consistent with the amended scheme and conventions.

Following the completion of primary coding for the dataset and finalisation of the codebook and conventions, a statistical quality control procedure was conducted to check the reliability of the data. This was conducted by randomly choosing 10% of the dataset’s 31 parent-level questions using a random number generator, for each jurisdiction (i.e. 3–4 parent-level questions per jurisdiction). An independent but legally-experienced coder (SW) was briefed on the project and provided with a copy of the protocol and coding conventions and coded these 25 questions blinded to SP’s and TN’s results. The divergence rate was zero.

Ethics approval was not required for this study as it was a review of publicly accessible government policy documents (laws), with no human participation or collection or use of any personal data.

### Data analysis

Composite summary variables that integrated multiple variables were developed to assess whether legal standards met evidence-based recommendations (where those were available). Scales were developed to capture the strength of legal support for built environment characteristics that did not have evidence-based standards. Results are reported in Tables 1, 2, 3 and 4 according to the variables analysed and scales developed, and in the text below to identify main differences between the jurisdictions for particular criteria.

**Table 1:** High-level elements addressing PA and related environments in Acts/Regulations

	NSW	VIC	TAS	WA	SA	NT	ACT	QLD
**Legislative objectives**								
1/Health in Planning Act	^□^	^○^	^✪^	^⊠^	^✪^	^⊠^	^⊠^	^✪^
2/Liveability in Planning Act	^□^	^⊠^	^□^	^⊠^	^✪^	^⊠^	^⊠^	^✪^
3/Public transport promotion in Transport Act	^□^	^✪^	^⊠^	^⊠^	^□^	^⊠^	^⊠^	^✪^
4/Active transport promotion in Transport Act	^⊠^	^✪^	^⊠^	^⊠^	^⊠^	^⊠^	^⊠^	^✪^
5/Private car use *reduction* in Transport Act	^⊠^	^✪^	^⊠^	^⊠^	^⊠^	^⊠^	^⊠^	^⊠^
7/Promotion of integrated land use and transport planning under Planning Act	^□^	^○^	^⊠^	^✪^	^✪^	^⊠^	^⊠^	^□^
8/Promotion of integrated land use and transport planning under Transport Act	^⊠^	^✪^	^⊠^	^⊠^	^⊠^	^⊠^	^⊠^	^✪^
**Climate and environment**								
9/Specific Climate Change Act	^□^	^✪^	^✪^	^□^	^✪^	^⊠^	^✪^	^⊠^
9.1/Climate impact of planning under Climate Act/ Regulations	NA	^○^	^□^	NA	^□^	NA	^□^	NA
10/Climate impact of planning under Planning Act	^⊠^	^✪^	^□^	^✪^	^✪^	^□^	^✪^	^✪^
11/Reducing environmental impact of transport under Transport Act	^□^	^✪^	^⊠^	^⊠^	^✪^	^⊠^	^⊠^	^✪^
**Public health intervention**								
6/Scope for public health intervention in planning in Public Health Act	^□^	^○^	^⊠^	^⊠^	^✪^	^⊠^	^✪^	^⊠^
6.1a/By enabling a Minister to require an assessment or inquiry into public health impact	NA	^○^	NA	NA	◊	NA	^✪^	NA
6.1b/By conferring a function on local councils to determine and respond to public health impact	NA	◊	NA	NA	^✪^	NA	◊	NA
**Number addressed out of 12 questions**^**a**^	0	11	2	2	7	0	3	7

^✪^Yes

^○^Yes with caution note

^⊠^No

^□^No with caution note

NA Not applicable, because this question was conditional on satisfying an affirmative answer to the preceding parent question

◊ Not selected as a response option (a different option/s selected instead)

*NSW* New South Wales, *VIC* Victoria, *TAS* Tasmania, *WA* Western Australia, *SA* South Australia, *NT* Northern Territory, *ACT* Australian Capital Territory, *QLD* Queensland

^a^The 12 questions comprise the 11 parent questions (1–11) and question 9.1

**Table 2:** Existence of standards in planning laws

	NSW	VIC	TAS	WA	SA	NT	ACT	QLD
**Density**								
12/Residential density target	^✪^	^✪^	^○^	^⊠^	^⊠^	^○^	^⊠^	^□^
13/Residential density target near activity centres	^⊠^	^✪^	^○^	^✪^	^✪^	^○^	^⊠^	^⊠^
14/Residential density target near public transit	^⊠^	^✪^	^○^	^⊠^	^✪^	^○^	^⊠^	^⊠^
***Planning law sets a residential density target***	Yes	Yes	Yes	Yes	Yes	Yes	No	No
**Access to destinations from homes**								
15/Distance from activity centre	^□^	^✪^	^○^	^✪^	^○^	^✪^	^○^	^⊠^
17/Distance from public open space	^✪^	^✪^	^⊠^	^□^	^○^	^✪^	^✪^	^✪^
18/Distance from primary schools	^⊠^	^✪^	^⊠^	^□^	^○^	^○^	^⊠^	^⊠^
***Strength of legal support for accessibility to destinations***^***a***^	Weak	Strong	Weak	Weak	Strong	Strong	Partial	Weak
**Support for active transport**								
19/Distance of homes from public transport	^✪^	^✪^	^✪^	^□^	^✪^	^✪^	^✪^	^□^
20/Car parking targets – max rate	^□^	^□^	^✪^	^○^	^✪^	^⊠^	^○^	^⊠^
21/Bicycle infrastructure	^□^	^✪^	^✪^	^✪^	^✪^	^✪^	^✪^	^✪^
***Strength of legal support for active transport***^***b***^	Weak	Partial	Strong	Partial	Strong	Partial	Strong	Weak
**Street block and footpaths**								
22/Street block size	^⊠^	^✪^	^⊠^	^✪^	^⊠^	^⊠^	^⊠^	^✪^
23/Footpath provision	^□^	^✪^	^□^	^□^	^⊠^	^⊠^	^⊠^	^✪^
***Strength of legal support for enabling pedestrian access***^***c***^	Weak	Strong	Weak	Partial	Weak	Weak	Weak	Strong
**Urban greening**								
25/Tree planting	^□^	^✪^	^⊠^	^✪^	^✪^	^○^	^⊠^	^✪^
26/Private open space landscaping	^✪^	^⊠^	^✪^	^○^	^✪^	^✪^	^✪^	^⊠^
27/Tree canopy cover	^□^	^✪^	^⊠^	^⊠^	^✪^	^⊠^	^⊠^	^⊠^
***Strength of legal support for urban greening***^***d***^	Weak	Partial	Weak	Partial	Strong	Partial	Weak	Weak
**Number of standards addressed (out of 12**^**e**^**)**	4	10	6	7	10	8	6	5
**Overall strength of legal support for standards**								
Overall number of categories with Strong legal support (out of 4 categories)	0	2	1	0	3	1	1	1
Overall number of categories with Partial legal support (out of 4 categories)	0	2	0	3	0	2	1	0
Overall number of categories with Weak legal support (out of 4 categories)	4	0	3	1	1	1	2	3

^✪^Yes

^○^Yes with caution note

^⊠^No

^□^No with caution note

*NSW* New South Wales, *VIC* Victoria, *TAS* Tasmania, *WA* Western Australia, *SA* South Australia, *NT* Northern Territory, *ACT* Australian Capital Territory, *QLD* Queensland

^a^Strength of legal support for accessibility to destinations classified as Strong if Yes to 15, 17, 18; Partial if Yes to two out of 15, 17, 18; Weak if Yes to one of 15, 17, 18 or No to all three

^b^Strength of legal support for active transport classified as Strong if Yes to 19, 20, 21; Partial if Yes to two out of 19, 20, 21; Weak of Yes to one of 19, 20, 21 or No to all three

^c^Strength of legal support for enabling pedestrian access classified as Strong if Yes to 22 and 23; Partial if Yes to either 22 or 23; Weak if No to both 22 and 23

^d^Strength of legal support for greening classified as Strong if Yes to 25, 26 and 27; Partial if Yes to two out of 25, 26 and 27; Weak if Yes to one of 25, 26 and 27 or No to all three

^e^The 12 standards comprise questions 12–14 (residential density) treated as one standard, and questions 15, 17–27

**Table 3:** Comparability to recommended built environment standards and criteria for active living

	Recommended standard (where available)	NSW	VIC	TAS	WA	SA	NT	ACT	QLD
**Minimum density targets**									
**Residential density**									
12.1a) Specifies target in terms of gross density		^✪^	^⊠^	^⊠^	NA	NA	^□^	NA	NA
12.1b) Minimum density target is ≥ 25 dwellings per hectare		^⊠^	^⊠^	^⊠^	NA	NA	^□^	NA	NA
**Residential density near activity centres**							^□^		
13.1a) Specifies target in terms of gross density		NA	^⊠^	^⊠^	^✪^	^⊠^	^□^	NA	NA
13.1b) Minimum density target is ≥ 25 dwellings per hectare		NA	^✪^	^✪^	^□^	^✪^	^⊠^	NA	NA
**Residential density near public transit**									
14.1a) Specifies target in terms of gross density		NA	^⊠^	^⊠^	NA	^⊠^	^□^	NA	NA
14.1b) Minimum density target is ≥ 25 dwellings per hectare		NA	^✪^	^✪^	NA	^✪^	^⊠^	NA	NA
***Planning law contains a minimum density target that meets the recommended standard***^***a***^	≥25 gross dwellings per hectare (Urban Liveability Checklist)	No	No	No	No	No	No	No	No
**Activity centres**									
**Location of homes from activity centres**									
15.1) Distance ≤ 800m		NA	^✪^	^✪^	^✪^	^✪^	^✪^	^✪^	NA
15.3) Specifies a % target of homes within ≤ 800m of an activity centre		NA	^○^	^⊠^	^□^	^○^	^⊠^	^⊠^	NA
15.3.1) Percentage target is ≥ 80%		NA	^✪^	NA	NA	^□^	NA	NA	NA
***Planning law contains an activity centre accessibility target that meets the recommended standard***^***b***^	≥ 80% of dwellings within 800m of a neighbourhood activity centre (Urban Liveability Checklist)	No	Yes	Partly	Partly	Partly	Partly	Partly	No
**Walkable catchment for activity centres**									
16) Specifies a walkable catchment area for activity centres		^✪^	^□^	^⊠^	^○^	^⊠^	^⊠^	^⊠^	^⊠^
16.1) Pedshed ≥ 0.60		^✪^	NA	NA	^✪^	NA	NA	NA	NA
***Planning law contains a walkable catchment target for activity centres that meets the recommended standard***^***c***^	Pedshed ≥ 0.60, calculated as the ratio of area within 800m street network buffer to the area within an 800m Euclidian (as the crow flies) buffer around a neighbourhood activity centre (Urban Liveability Checklist)	Yes	No	No	Yes	No	No	No	No
**Promotion of active frontage in activity centres**									
24) Expressly promotes active frontage in activity centres		^✪^	^✪^	^✪^	^✪^	^✪^	^○^	^✪^	^⊠^
*Crime prevention through environmental design features*									
24.1a) Non-residential ground floor use		^✪^	^✪^	◊	^✪^	^✪^	◊	^✪^	NA
24.1b) Limits to blank walls		^✪^	◊	^✪^	^✪^	◊	^✪^	◊	NA
24.1c) Transparency of window coverings, fencing		◊	^✪^	^✪^	^✪^	^✪^	^✪^	◊	NA
24.1d) Coverage of windows, entrances or shopfronts		◊	◊	^✪^	◊	^✪^	◊	◊	NA
24.1e) Limits to height of fences or walls		◊	^✪^	^✪^	◊	◊	◊	◊	NA
*Minimising conflict with vehicles*									
24.1f) Parking away from street frontage		^✪^	^✪^	^✪^	◊	^✪^	◊	^✪^	NA
24.1g) Limits to services at street level		◊	^✪^	◊	^✪^	◊	^✪^	◊	NA
*Convenience, comfort and interest*									
24.1h) Direct pedestrian access		^✪^	^✪^	^✪^	◊	^✪^	^✪^	◊	NA
24.1i) Provision of awnings/shelter		^✪^	^✪^	^✪^	^✪^	^✪^	^✪^	◊	NA
24.1j) Limits to building setbacks		^✪^	◊	◊	◊	◊	◊	◊	NA
24.1k) Alfresco dining		◊	◊	◊	◊	^✪^	^✪^	◊	NA
***Strength of legal support for promoting active frontage in activity centres***^***d***^		Strong	Strong	Strong	Strong	Strong	Strong	Partial	Weak
**Access to public open space**									
**Availability of distance standards for the location of homes from public open space**									
17.1a) For local/neighbourhood parks		^□^	^✪^	^⊠^	^⊠^	^⊠^	^✪^	^✪^	^✪^
17.1b) For active open space		^⊠^	^✪^	^⊠^	^⊠^	^⊠^	^⊠^	^⊠^	^⊠^
17.1c) For linear park/open space corridor		^⊠^	^✪^	^⊠^	^⊠^	^⊠^	^⊠^	^⊠^	^⊠^
17.1d) For district parks		^□^	^⊠^	^⊠^	^⊠^	^⊠^	^⊠^	^⊠^	^□^
17.1e) For district sport precincts		^⊠^	^⊠^	^⊠^	^⊠^	^⊠^	^⊠^	^⊠^	^□^
17.1f) For regional/metropolitan parks		^□^	^⊠^	^⊠^	^⊠^	^⊠^	^⊠^	^⊠^	^⊠^
17.1g) For regional/metropolitan sporting precincts		^⊠^	^⊠^	^⊠^	^⊠^	^⊠^	^⊠^	^⊠^	^⊠^
17.1h) For public open space generally		^✪^	^○^	^⊠^	^⊠^	^✪^	^✪^	^⊠^	^✪^
**Local/neighbourhood park**									
17.2) Location of homes ≤ 400m from a local/neighbourhood park		NA	^✪^	NA	NA	NA	^✪^	^✪^	^✪^
17.2.2) ≥ 80% of homes within the specified distance of a local/neighbourhood park		NA	^✪^	NA	NA	NA	NA	^✪^	NA
17.3) Specifies a minimum size dimension for a local/neighbourhood park		NA	^✪^	NA	NA	NA	^⊠^	^⊠^	^□^
17.3.1) Minimum size dimension of local/neighbourhood park ≥ 1.5ha		NA	^□^	NA	NA	NA	NA	NA	NA
17.4) Specifies other design criteria for local/neighbourhood parks		NA	^✪^	NA	NA	NA	^⊠^	^✪^	^□^
**Active open space**									
17.5) Location of homes ≤ 400m from active open space		NA	^⊠^	NA	NA	NA	NA	NA	NA
17.5.2) ≥ 80% of homes within the specified distance of active open space		NA	^✪^	NA	NA	NA	NA	NA	NA
17.6) Specifies a minimum size dimension for active open space		NA	^✪^	NA	NA	NA	NA	NA	NA
17.6.1) Minimum size dimension for active open space ≥ 1.5ha		NA	^✪^	NA	NA	NA	NA	NA	NA
17.7) Specifies other design criteria for active open space		NA	^✪^	NA	NA	NA	NA	NA	NA
**Linear parks/trails**									
17.8) Location of homes ≤ 400m from linear parks/trails		NA	^⊠^	NA	NA	NA	NA	NA	NA
17.8.2) ≥ 80% of homes within the specified distance of linear parks/trails		NA	^✪^	NA	NA	NA	NA	NA	NA
17.9) Specifies other design criteria for linear parks/trails		NA	^✪^	NA	NA	NA	NA	NA	NA
**Public open space**									
17.22) Location of homes within ≤ 400m from public open space		^✪^	^⊠^	NA	NA	^✪^	^✪^	NA	^✪^
17.22.2) ≥ 80% of homes within specified distance of public open space		^✪^	^✪^	NA	NA	^□^	NA	NA	NA
17.23) Specifies a minimum size dimension for public open space		^⊠^	^✪^	NA	NA	^✪^	^⊠^	NA	^⊠^
17.23.1) Minimum size dimension of public open space ≥ 1.5ha		NA	^⊠^	NA	NA	^⊠^	NA	NA	NA
17.24) Specifies other design criteria for public open space		^□^	^✪^	NA	NA	^✪^	^⊠^	NA	^⊠^
***Planning law contains an accessibility target for public open space that meets the recommended standard***^***e***^	≥ 80% of dwellings ≤ 400m of ≥ 1.5 ha of open space (Urban Liveability Checklist)	Partly	Partly	No	No	Partly	Partly	Partly	Partly
**Schools**									
18.1) Location of homes ≤ 800m from a government primary school		NA	^✪^	NA	NA	^⊠^	^✪^	NA	NA
18.3) ≥ 80% of dwellings within the specified distance of a government primary school		NA	^⊠^	NA	NA	^□^	NA	NA	NA
***Planning law contains an accessibility target for primary schools that meets the recommended standard***^***f***^	≥ 80% of dwellings ≤ 800m from a government primary school (Urban Liveability Checklist)	No	Partly	No	No	No	Partly	No	No
**Public transport**									
**Access to bus services**									
19.1a) Location of homes ≤ 400m from a bus stop		^✪^	^✪^	NA	NA	^✪^	^✪^	^□^	NA
19.1.2a) Target of ≥ 80% homes within the specified distance of bus services		NA	^✪^	NA	NA	^□^	NA	^✪^	NA
19.1.4a) Frequency of bus services specified, at least every 30 mins		^✪^	NA	NA	NA	^✪^	NA	NA	NA
**Train services**									
19.1b) Location of homes ≤ 800m from a train stop		^○^	^✪^	NA	NA	^✪^	NA	NA	NA
19.1.2b) Target of ≥ 80% homes within the specified distance of train services		NA	^✪^	NA	NA	^□^	NA	NA	NA
19.1.4b) Frequency of train services specified, at least every 30 mins		^✪^	NA	NA	NA	NA	NA	NA	NA
**Tram services**									
19.1c) Location of homes ≤ 600m from a tram stop		NA	^✪^	NA	NA	^⊠^	NA	NA	NA
19.1.2c) Target of ≥ 80% homes within the specified distance of tram services		NA	^✪^	NA	NA	^□^	NA	NA	NA
19.1.4c) Frequency of tram services specified, at least every 30 mins		NA	NA	NA	NA	NA	NA	NA	NA
**Public transport generally**									
19.1d) Location of homes ≤ 400m of a public transport stop		NA	^✪^	^⊠^	NA	^✪^	^✪^	NA	NA
19.1.2d) Target of ≥ 80% homes within the specified distance of public transport		NA	^✪^	NA	NA	^□^	NA	NA	NA
19.1.4d) Frequency of public transport services specified, at least every 30 mins		NA	NA	NA	NA	NA	^⊠^	NA	NA
***Planning law contains an accessibility target for public transport that meets the recommended standard***^***g***^	80% of dwellings located within at least one of: • ≤ 400m from a bus stop with a scheduled service every 30 mins 7am-7pm on a normal weekday • ≤ 600m from a tram stop with a scheduled service every 30 mins 7am-7pm on a normal weekday • ≤ 800m from a train stop with a scheduled service every 30 mins 7am-7pm on a normal weekday (Urban Liveability Checklist)	Partly	Partly	No	No	Partly	Partly	Partly	No
**Demand management**									
20a) Specifies a maximum car parking rate		^□^	^□^	^✪^	^○^	^✪^	^⊠^	^○^	^⊠^
20b) Specifies a minimum car parking rate		^✪^	^✪^	^✪^	^✪^	^✪^	^✪^	^✪^	^□^
20.1) Where a minimum car parking rate is set, allows for reduction of this provision		^○^	^✪^	^✪^	^✪^	^✪^	^✪^	^✪^	NA
***Strength of legal support for discouraging car usage***^***h***^		Partial	Partial	Strong	Strong	Strong	Partial	Strong	Weak
**Bicycle infrastructure**									
*Bicycle parking*									
21) Sets standards for the provision of bicycle parking		^□^	^✪^	^✪^	^✪^	^✪^	^✪^	^✪^	^✪^
21.1.1a) Bicycle parking for residential		NA	^✪^	^✪^	^✪^	^✪^	^✪^	^✪^	◊
21.1.1b) Bicycle parking for education		NA	^✪^	^✪^	◊	^✪^	◊	^✪^	^✪^
21.1.1c) Bicycle parking for retail		NA	^✪^	^✪^	◊	^✪^	^✪^	^✪^	^✪^
21.1.1d) Bicycle parking for offices		NA	^✪^	^✪^	◊	^✪^	^✪^	^✪^	^✪^
21.1.1e) Bicycle parking for healthcare		NA	^✪^	^✪^	◊	^✪^	◊	^✪^	^✪^
21.1.1f) Bicycle parking for sport and recreation		NA	^✪^	^○^	◊	^✪^	◊	◊	◊
21.1.1g) Bicycle parking for public transport		NA	◊	^○^	◊	◊	◊	◊	◊
*End-of-trip facilities (EoT) (lockers and/or showers)*									
21.1a) Sets standards for the provision of showers		^□^	^✪^	^⊠^	^⊠^	^⊠^	^✪^	^○^	^✪^
21.1b) Sets standards for the provision of lockers		^□^	^✪^	^⊠^	^⊠^	^⊠^	^✪^	^✪^	^✪^
21.1.2a) EoT for residential		NA	^✪^	NA	NA	NA	^✪^	^✪^	◊
21.1.2b) EoT for education		NA	^✪^	NA	NA	NA	◊	^✪^	^✪^
21.1.2c) EoT for retail		NA	^✪^	NA	NA	NA	^✪^	^✪^	^✪^
21.1.2d) EoT for offices		NA	^✪^	NA	NA	NA	^✪^	^✪^	^✪^
21.1.2e) EoT for healthcare		NA	^✪^	NA	NA	NA	◊	^✪^	^✪^
21.1.2f) EoT for sport and recreation		NA	^✪^	NA	NA	NA	◊	◊	◊
*Bicycle paths*									
21.1) Sets standards for the provision of bicycle paths		^⊠^	^✪^	^⊠^	^⊠^	^⊠^	^⊠^	^⊠^	^⊠^
***Strength of legal support for bicycle infrastructure***^***i***^		Weak	Strong	Partial	Partial	Partial	Partial	Partial	Partial
**Street blocks and footpaths**									
**Street blocks**									
22.1.1) Specifies a block length, where upper limit is ≤150m		NA	^⊠^	NA	^⊠^	NA	NA	NA	^□^
22.1.3) Specifies a block width, where upper limit is ≤150m		NA	^✪^	NA	^✪^	NA	NA	NA	NA
22.1.5) Specifies a block perimeter ≤ 600m		NA	^✪^	NA	^✪^	NA	NA	NA	NA
***Planning law contains street block standards that meet recommendation***^***j***^	Blockfaces of 110m or less; blocks should not exceed 150m (ITDP guidance)	No	Yes	No	Yes	No	No	No	No
**Footpaths**									
23.1a) Standards address width of footpath		NA	^✪^	NA	NA	NA	NA	NA	◊
23.1b) Standards specify the sides of the street where footpath is required		NA	^✪^	NA	NA	NA	NA	NA	^✪^
23.1c) Standards require increased footpath provision near schools, shops or transit		NA	^✪^	NA	NA	NA	NA	NA	◊
***Strength of legal support for footpath provision***^***k***^		Weak	Strong	Weak	Weak	Weak	Weak	Weak	Partial
**Urban greening**									
**Provision of trees**									
25.1a) Standards address spacing of trees		NA	^✪^	NA	◊	^✪^	◊	NA	^✪^
25.1b) Standards address placement of trees		NA	◊	NA	^✪^	◊	^✪^	NA	^✪^
25.1c) Standards address size of trees		NA	^✪^	NA	^✪^	^✪^	◊	NA	◊
25.1d) Standards address canopy cover of trees		NA	◊	NA	^✪^	^✪^	◊	NA	◊
25.1e) Standards address number of trees		NA	^✪^	NA	^✪^	^✪^	^✪^	NA	^✪^
**Private open space for gardens, planting, soft landscaping**									
26.1a) Standards expressed as minimum % site for planting		^✪^	NA	◊	^✪^	^✪^	◊	^○^	NA
26.1b) Standards expressed as minimum dimensions for planting		^✪^	NA	^○^	^✪^	^✪^	^✪^	◊	NA
26.1c) Standards expressed as maximum % site coverage for buildings		◊	NA	^✪^	◊	◊	◊	◊	NA
**Tree canopy cover **									
27) Specifies a % target for increasing tree canopy cover		^□^	^✪^	^⊠^	^⊠^	^✪^	^⊠^	^⊠^	^⊠^
***Strength of legal support for urban greening***^***l***^		Weak	Partial	Weak	Partial	Strong	Partial	Weak	Weak
**Overall assessment**									
**Standards meeting recommendations**									
Overall number of standards Meeting recommendations (out of 7 available)^m^		1	2	0	2	0	0	0	0
Overall number of standards Partly meeting recommendations (out of 7 available)		2	3	1	1	3	4	3	1
Overall number of standards Not meeting recommendations (including where not addressed) (out of 7 available)		4	2	6	4	4	3	4	6
**Extent of legal support for other categories**^**n**^									
Overall number of categories with Strong legal support (out of 5 categories)		1	3	2	2	3	1	1	0
Overall number of categories with Partial legal support (out of 5 categories)		1	2	1	2	1	3	2	2
Overall number of categories with Weak legal support (out of 5 categories)		3	0	2	1	1	1	2	3

^✪^Yes

^○^Yes with caution note

^⊠^No

^□^No with caution note

NA Not applicable, because this question was conditional on satisfying an affirmative answer to the preceding parent question

◊Not selected as a response option (a different option/s selected instead)

*NSW* New South Wales, *VIC* Victoria, *TAS* Tasmania, *WA* Western Australia, *SA* South Australia, *NT* Northern Territory, *ACT* Australian Capital Territory, *QLD* Queensland

Urban Liveability Checklist is the ‘The Healthy Liveable Communities Urban Liveability Checklist’ developed by RMIT University in 2019; ITDP guidance refers to the recommendations set out in the ‘Pedestrians First’ tool developed by the Institute for Transportation & Development Policy) in 2018

^a^Minimum density target classified as Yes (meeting the recommended standard) if Yes to 12.1a and 12.1b, 13.1a and 13.1b, and/or 14.1a and 14.1b (i.e. density target was a gross measure and ≥ 25 dwellings per hectare). Classified as No (not meeting) if the density target was not expressed as a gross measure, even if it was ≥ 25 dwellings per hectare

^b^Activity centre accessibility target classified as Yes (meeting the recommended standard) if Yes to 15.1 and 15.3.1 (i.e. ≥ 80% of dwellings within 800 m of any type of activity centre); Partly meeting if Yes to 15.1 but No/NA to 15.3.1 (i.e. meets the distance standard but not the percentage of dwellings component). Classified as No (not meeting) if NA/No to 15.1 and 15.3.1

^c^Walkable catchment target classified as Yes (meeting the recommended standard) if Yes to 16 and 16.1. Classified as No (not meeting) if No/NA to 16 and 16.1. Note that the radius used by NSW and WA was generally ≤ 400 m (i.e. less than the 800 m specified in the recommended standard, although WA used 800 m for strategic centres)

^d^Strength of legal support for active frontage in activity centres classified as Strong if the jurisdiction addressed at least one of the design features in each category (i.e. crime prevention through environmental design features (24.1a-e); minimising conflict with vehicles (24.1f-g); convenience, comfort and interest (24.1 h-k)); Partial if it addressed one or two of the categories; Weak if it did not expressly address active frontage in activity centres (i.e. No to 24)

^e^Public open space accessibility target classified as Yes if meeting all elements of the recommended standard (i.e. percentage (≥ 80% dwellings), distance (≤ 400 m) and size of open space (≥ 1.5 ha)); Partly if meeting at least one of the three elements; and No if it did not have any distance standards for the location of homes from public open space

^f^Primary school accessibility target classified as Yes if meeting all elements of the recommended standard (i.e. Yes to 18.3 (≥ 80% dwellings) and Yes to 18.1 (distance ≤ 800 m); Partly if Yes to 18.1 only; No if NA to 18.1 (i.e. did not have any distance standards for the location of homes from a primary school)

^g^Public transport accessibility target classified as Yes if meeting all elements of the recommended standard for at least one form of public transport (i.e. percentage (≥ 80% dwellings), distance (≤ 400 m from bus stop; ≤ 600 m from tram stop; ≤ 800 m from train stop) and frequency (at least every 30 min)); Partly if meeting at least one of the elements for at least one form of public transport; No if not addressing or meeting any of the elements of the recommended standard for any form of public transport

^h^Strength of legal support for discouraging car usage classified as Strong if Yes to 20a (i.e. specifies a maximum rate); Partial if No to 20a but Yes to 20.1 (i.e. no maximum rate but allows for reduction of minimum car parking rate); Weak if neither was addressed

^i^Strength of legal support for bicycle infrastructure classified as Strong if Yes to 21, 21.1a, 21.1b and 21.1 (i.e. sets standards for bicycle parking, end-of-trip facilities, and bicycle paths); Partial if only sets standards for bicycle parking and/or end-of-trip facilities; Weak if it did not address any of these standards

^j^Street block standards classified as meeting recommendation if Yes to 22.1.5 (perimeter of ≤ 600 m was considered to be equivalent to the ITDP recommendation of blocks not exceeding 150 m); and/or Yes to both 22.1.1 and 22.1.3 (i.e. meeting the recommendations for block length and width)

^k^Strength of legal support for footpath provision classified as Strong if Yes to 23.1a-c (i.e. addressing width, sides and increased provision near schools, shops or transit); Partial if addressing one or two of these elements (i.e. Yes to one or two out of 23.1a-c); Weak if not addressing any of these elements for footpath provision

^l^Strength of legal support for urban greening classified as Strong if addressing at least one aspect of each of the three categories (i.e. Yes to any of 25.1a-e (provision of trees); Yes to any of 26.1a-c (private open space); and Yes to 27 (tree canopy cover)); Partial if addressing two out of the three categories; Weak if addressing one of the three categories or none at all

^m^The standards with recommendations are for: residential density (12.1–14.1), activity centre accessibility (15.1–15.3.1), walkable catchment area for activity centres (16–16.1), public open space accessibility (17.1a-17.24), primary school accessibility (18.1, 18.3), public transport accessibility (19.1a-19.1.4c), street block size (22.1.1–22.1.5)

^n^Extent of legal support are for: promoting active frontage in activity centres (24–24.1 k), demand management (20a-20.1), bicycle infrastructure (21–21.1), footpath provision (23.1a-c), and urban greening (25.1a-27)

**Table 4:** Implementation and monitoring of planning laws

	NSW	VIC	TAS	WA	SA	NT	ACT	QLD
**Jurisdiction-wide provisions**								
28/ A single source of default, comprehensive provisions applying jurisdiction-wide for design and planning	^⊠^	^✪^	^✪^	^⊠^	^✪^	^✪^	^✪^	^⊠^
**Design review**								
30/Addresses design review panels	^✪^	^○^	^⊠^	^✪^	^✪^	^⊠^	^✪^	^⊠^
30a/By requiring establishment of design review panels	◊	◊	NA	◊	◊	NA	^○^	NA
30b/By allowing the establishment of design review panels	^✪^	◊	NA	◊	^✪^	NA	◊	NA
30c/By allowing the establishment of committees (but not design review panels specifically)	◊	^○^	NA	^✪^	◊	NA	◊	NA
30.1/Specifies circumstances where design review is required	^✪^	^⊠^	NA	^□^	^⊠^	NA	^✪^	NA
***Strength of addressing design review***^***a***^	Strong	Partial	Weak	Partial	Partial	Weak	Strong	Weak
**Monitoring and enforcement**								
29/Responsibility for enforcement	^○^	^✪^	^✪^	^✪^	^⊠^	^✪^	^⊠^	^⊠^
31/Performance monitoring	^✪^	^□^	^✪^	^⊠^	^✪^	^□^	^⊠^	^⊠^
31.1a/Allows Minister to set targets	◊	NA	◊	NA	^✪^	NA	NA	NA
31.1b/Allows a Minister to appoint a responsible authority for evaluation	◊	NA	^✪^	NA	◊	NA	NA	NA
31.1c/ Specifies responsibility for evaluation	^✪^	NA	^✪^	NA	^✪^	NA	NA	NA
31.1d/Sets out obligations for reporting	◊	NA	^✪^	NA	^✪^	NA	NA	NA
31.1e/Requires statutory instruments to address reporting obligations	^✪^	NA	◊	NA	◊	NA	NA	NA
***Strength of addressing enforcement and monitoring***^***b***^	Strong	Partial	Strong	Partial	Partial	Partial	Weak	Weak
**Overall strength of addressing implementation and monitoring**^**c**^	Partial	Partial	Partial	Weak	Partial	Partial	Partial	Weak

^✪^Yes

^○^Yes with caution note

^⊠^No

^□^No with caution note

NA Not applicable, because this question was conditional on satisfying an affirmative answer to the preceding parent question

◊Not selected as a response option (a different option/s selected instead)

*NSW* New South Wales, *VIC* Victoria, *TAS* Tasmania, *WA* Western Australia, *SA* South Australia, *NT* Northern Territory, *ACT* Australian Capital Territory, *QLD* Queensland

^a^Strength of addressing design review classified as Strong if Yes to 30 (i.e. addresses design review panels), and 30.1 (specifies circumstances where design review is required); Partial if Yes to 30 and No to 30.1 (i.e. addresses design review panels but does not make design review mandatory in any circumstances); Weak if No to 30 (i.e. does not address design review panels)

^b^Strength of addressing enforcement and monitoring classified as Strong if Yes to 29 (responsibility for enforcement) and 31 (performance monitoring); Partial if Yes to 29 (responsibility for enforcement) or 31 (performance monitoring); Weak if No to both 29 (responsibility for enforcement) and 31 (performance monitoring)

^c^Overall strength of addressing implementation and monitoring classified as *Strong* if Yes to 28 (single source of default comprehensive provisions applying jurisdiction-wide for design and planning) and two Strong ratings for design review and enforcement and monitoring; *Partial* if No to 28 and two Strong ratings for design review and enforcement and monitoring, Yes to 28 and at least one Strong/Partial rating for design review and enforcement and monitoring; *Weak* if No to 28 and two Weak/Partial ratings, or Yes to 28 and two Weak ratings for design review and enforcement and monitoring

## Results

### High-level legislative support

The extent of high-level legislative support for PA is presented in Table 1. High-level support for walking and cycling was demonstrated by the provision of objectives in Planning and Transport Acts that expressly promote health, liveability, public and active transport, reduced private car use reduction, and integrated land use and transport planning; statutory obligations to consider the climate or environmental impact of planning and transport; and public health functions or powers to intervene in planning.

Only three of the eight Australian jurisdictions addressed the majority of the high-level elements for PA and related environments in terms of legislative objectives relevant to walking and cycling in Planning and Transport Acts, legislative requirements to consider the climate impact of transport and planning, and scope for public health intervention in urban planning under the Public Health Act (Table 1). In terms of legislative objectives, health as a planning objective was the most widely addressed (addressed by half of the jurisdictions). In two of these jurisdictions (South Australia (SA), Queensland (QLD)), ecological sustainability formed part of the overarching objective of the Planning Act and guiding principles to achieve this were set out that addressed the creation of healthy communities (Planning, Development and Infrastructure Act 2016 (SA), Sects. 12(1) and 14(d)(ii); Planning Act 2016 (QLD), Sects. 3(1)-(3), 3(3)(c)(i)). SA and QLD were also the only two jurisdictions that expressly mentioned liveability as an objective of their Planning Acts.

In Tasmania (TAS), the health objective was framed in terms of promoting the health and wellbeing of Tasmanians by ‘ensuring a pleasant, efficient and safe environment for working, living and recreation’ (Land Use Planning and Approvals Act 1993 Sch 1, Part 2 clause (f)). In Victoria (VIC), the health objective was incorporated into the Planning Act via ‘interface legislation’ which brought planning authorities within the scope of the Transport Integration Act 2010 (VIC) and the obligation to consider the transport system objective of supporting health and wellbeing. This obligation applied to the extent that planning functions were likely to have a significant impact on the ‘transport system’ (defined to include public transport networks, cycling paths and footpaths); there was otherwise no obligation to promote human health in planning (Planning and Environment Act 1987 (VIC), Sect. 3A; Transport Integration Act 2010 (VIC), Sects. 13, 25). In other jurisdictions, human health was not mentioned as a planning objective, or only construed in narrow terms in the context of promoting the proper construction and maintenance of buildings (Environmental Planning and Assessment Act 1979 (New South Wales (NSW)), Sect. 1.3(h)).

In terms of the Public Health Act, only three jurisdictions set out provisions that enabled public health intervention in planning. These were general provisions that were not specific to planning, except in SA which conferred a function on local municipal councils to assess development in its area to determine and respond to public health impacts or potential public health impacts (Public Health Act 2011 (SA), Sect. 37(1)(g)).

Transport objectives to increase active and public transport were addressed by two jurisdictions (VIC, QLD). For example, in VIC this was through objectives to promote forms of transport which have the least impact on the environment and reduce overall contribution of greenhouse gas emissions, and which have the greatest benefit for and least negative impact on health and wellbeing, as well as objectives for the Head of Transport for Victoria to increase the share of public transport, walking and cycling trips and actively promote public transport as an alternative to motor car travel (Transport Integration Act 2010 (VIC), Sects. 10, 13, 64B).

In QLD, this was through objectives to ensure that public transport offers an attractive alternative to private transport, promote urban development that maximises the use of public transport, increase opportunities for people to access public transport by walking and cycling, and ensure there is supportive development and infrastructure for active transport (Transport Planning and Coordination Act 1994 (QLD), Sect. 8A). Such objectives were not mentioned in the Transport Acts of other jurisdictions, or only considered to a limited extent or less explicit manner; for example by requiring a pricing tribunal to consider the impact of a maximum fare recommendation or determination on the use of public transport and the need to increase the proportion of travel undertaken by sustainable modes (Passenger Transport Act 2014 (NSW), Sect. 124(3)(e)), or by expressing an objective of creating a ‘passenger transport network’ that encourages transport choices that minimise harm to the environment (where ‘passenger transport network’ was not specific to public transport but included paid carriage of passengers by motor vehicles) (Passenger Transport Act 1994 (SA), Sect. 3).

The promotion of integrated transport and land use planning was addressed in legislation by three jurisdictions under their Planning Act (SA, VIC, Western Australia (WA)), and under the Transport Act in two jurisdictions (VIC, QLD). For example, this was addressed in SA by setting out integrated delivery principles that required the coordination of policies (including those outside the planning system) and the promotion of integrated transport connections in planning, design and development (Planning, Development and Infrastructure Act 2016 (SA), Sect. 14). It was addressed in WA by conferring functions on the WA Planning Commission to plan for the coordinated provision of transport and infrastructure for land development (Planning and Development Act 2005 (WA), Sect. 14). Such objectives were not regarded as addressed where the legislation referred to integrated systems of transport or land use, but not in conjunction or considering their interactions with each other, or if the objectives were not addressed in legislation (i.e. the Planning or Transport Acts) but under other statutory instruments (e.g. in NSW, integrated land use and transport planning was addressed under a Ministerial Direction issued under the Environmental Planning and Assessment Act 1979 (NSW)).

Half of the jurisdictions had a Climate Change Act (Australian Capital Territory (ACT), SA, TAS, VIC). However, the obligations to consider the climate impact of planning were mainly addressed under the Planning Act rather than under the climate legislation. It was only specifically addressed by the Climate Act in one jurisdiction through the requirement to prepare an adaptation action plan for the built environment and transport system (VIC), although this Act also contained an obligation to consider climate impact in decisions under other Acts listed in the Schedule, which did not cover the Planning Act. In some of the other jurisdictions, the Climate Act only contained general provisions that, if exercised in relation to planning, could have implications for this sector; for example, by allowing the making of sector targets (e.g. TAS, SA, ACT) and/or a Minister to recommend amendments to a law if reasonably necessary to achieve the objects of the Climate Act (e.g. ACT, SA).

### Existence of standards in planning laws

The extent to which the planning laws of each jurisdiction contained measurable standards for density, destination and public transport accessibility, active transport infrastructure and demand reduction, street block and footpath provision, and urban greening, is set out in Table 2. An example of a measurable standard, in the case of active transport infrastructure, is where the jurisdiction sets out minimum rates of bicycle parking to be provided in specified facilities (e.g. schools, offices), but not where they have simply recommended that bicycle storage facilities be provided.

Four of the eight jurisdictions addressed more than half of the 12 standards (VIC, WA, SA, NT), and these were the same jurisdictions that demonstrated strong/partial legal support for at least three out of the four grouped categories (i.e. destination accessibility, support for active transport, enabling pedestrian access, and urban greening). The standards that were most widely addressed were for a residential density target, distance of homes from an activity centre, distance of homes from public open space, distance of homes from public transport, provision of bicycle infrastructure, and provision of private open space landscaping, each of which was addressed by six out of the eight jurisdictions; and provision of bicycle infrastructure which was addressed by seven of the jurisdictions. The least commonly addressed standards were for street block size (addressed by three jurisdictions), and footpath provision and tree canopy cover (each addressed by two jurisdictions).

In terms of the grouped categories (i.e. destination accessibility, support for active transport, street blocks and footpaths, urban greening), legal support was most widespread for active transport with strong/partial support among six jurisdictions. However, only three or four jurisdictions demonstrated strong/partial legal support for each of the other domains (i.e. accessibility to destinations, enabling pedestrian access, and urban greening).

### Comparability to recommended standards and other elements addressed

Table 3 compares existing standards in the planning laws to recommended standards where those were available (i.e. for minimum density targets, activity centre accessibility, walkable catchment target, access to public open space, access to primary schools, access to public transport and street block size). It also identifies the strength of legal support for other matters for which recommended standards were unavailable (i.e. active frontage promotion, demand management, provision of bicycle infrastructure, footpath provision and urban greening).

Only three jurisdictions had at least one standard in their planning law that fully met available recommendations, and these were for activity centre accessibility, walkable catchment and street block size. An example of a standard that fully met the recommendation for activity centre accessibility (i.e. at least 80% of dwellings within 800 m of a neighbourhood activity centre [34]) was in the Victorian Precinct Structure Planning Guidelines 2021, which stated that ‘80–90% of dwellings should be located within 800 m of an activity centre’ (Part 3, T19). An example of where it was partially met was in Element 7 (O4) of the Western Australian Liveable Neighbourhoods 2009 (the only part of Liveable Neighbourhoods with legal effect), which stated that a ‘substantial majority’ (i.e. not a specified percentage) of dwellings should be within a 400 to 500 m radius of a neighbourhood centre. Most jurisdictions which addressed this standard, only partly met the recommendation because they did not specify a percentage target of dwellings that were to be located within the stated distance.

There were no jurisdictions that had planning law standards that fully met the recommendations for a minimum residential density target, public open space accessibility, primary school accessibility and public transport accessibility. In terms of minimum residential density targets, the main reason why jurisdictions did not fully meet recommendations was because they used net density rather than gross density. The latter is a more conservative measure than net density as it includes non-residential uses such as parks and schools [37, 38]. Of the jurisdictions that used gross density (NSW, WA), the minimum residential density target was below the recommended threshold of at least 25 dwellings per hectare [34].

In terms of public open space accessibility, distances from dwellings were provided for local/neighbourhood parks in four jurisdictions, and public open space (generally described) in five jurisdictions. Of those jurisdictions which set out such a standard, most only partly met the recommendation (i.e. at least 80% of dwellings within 400 m of at least 1.5 hectares of open space [34]). VIC was the closest to meeting all components of the recommendation but did not meet the size component for local/neighbourhood parks which it specified should generally be 1 hectare in area (if not designed to include active open space), or the distance component for active open space which it specified should be within 1 km of dwellings (Victorian Planning Provisions VPP56.05–2, Standard C13). Of the six jurisdictions that had a distance standard for any type of public open space, only three set out design criteria for these spaces (e.g. in relation to being fit for purpose, connectivity, location, diversity/adaptability, features/facilities, shape and boundaries, and shade).

Only three jurisdictions established a distance standard for the location of homes from primary schools, two of which met the recommendation (i.e. within 800 m), and one of which also specified a percentage target of dwellings (70%) (although that fell short of the recommended threshold of 80% of dwellings (Precinct Structure Planning Guidelines 2021, Part 3, T18)). In relation to public transport accessibility, a distance standard was provided in six jurisdictions, and most commonly for bus services compared to other modes. While most of these jurisdictions set out distances within the scope of the recommendation (e.g. within 400 m of a bus stop), they did not fully address the recommendation because they either did not specify or meet the recommended percentage target of dwellings with this access, or did not specify the frequency of these services.

For other built environment characteristics without recommended standards to compare against, there was strong legal support for active frontage promotion in six jurisdictions and for demand management in half of the eight jurisdictions. To be classified as providing strong legal support for demand management (i.e. strategies that make walking, cycling and public transport use more attractive and motor vehicle usage less attractive [39]), jurisdictions needed to set out maximum parking rates. This was achieved to varying degrees, and there was no jurisdiction that only set maximum rates. For example, ACT mostly set out a minimum parking rate but established a maximum rate of 3 spaces per 100m^2^ for mixed use developments greater than 1000m^2^ in a particular city centre zone (Territory Plan 2008, Part 11.1, clause 3.2.2). The maximum parking rate in TAS (expressed as no onsite parking or not above existing numbers), was limited to areas that were subject to a parking precinct plan (Tasmanian Planning Scheme State Planning Provisions, C2.7.1(A1)). Maximum parking rates in WA were only set out for residential apartments, the limit being double the minimum number of parking bays otherwise specified (State Planning Policy 7.3 Residential Design Codes (volume 2) 2019, Element 3.9, A3.9.3). By contrast, SA set out maximum rates for multiple development types (e.g. non-residential development and tourist accommodation, but not residential) in multiple zones (e.g. city, urban corridors, and suburban activity centre zones) (Planning and Design Code, Part 4 (General Development Policies), Transport, Access and Parking, PO5.1 and DTS/DPF5.1 (Vehicle parking rates)).

Legal support for bicycle infrastructure was commonly only partial (in six out of the eight jurisdictions), weak for footpath provision (in six jurisdictions) and weak/partial for urban greening (in seven jurisdictions). Six jurisdictions only had partial support for bicycle infrastructure because while half of these set standards for both bicycle parking and end-of-trip facilities, none of these set standards for the provision of bicycle paths. The jurisdictions that set standards for bicycle parking did so by setting out minimum bicycle parking rates for multiple types of facilities (e.g. residential, retail and offices, and less commonly in healthcare, sport and recreation, and public transport facilities). VIC was the only jurisdiction to set standards for bicycle path provision by requiring cycle paths for residential subdivision, according to criteria for different street types (e.g. for arterial roads, a 3 m wide shared foot and cycle path on each side (Victorian Planning Provisions VPP56.06–5 Standard C18, VPP56.06–7 Standard C20, Table C1)).

Legal support for footpath provision was mainly weak because jurisdictions did not set out any standards for this, except in QLD and VIC. The QLD standard (which applied to reconfigurations of a residential lots involving road modifications) required a footpath on at least one side of a new road if that was used mainly to provide direct access to a created lot, or on both sides for another new road (Planning Regulation 2017, Sch 12A clause 7). In VIC, residential subdivisions were subject to standards that required footpaths to be provided according to criteria for different street types (e.g. for connector streets, a 1.5 m footpath on both sides, widened to 2 m in the vicinity of a school, shop or other activity centre (Victorian Planning Provisions VPP56.06–5, Standard C18; Table C1)). There was also a performance target in the VIC Precinct Structure Planning Guidelines 2021 for the provision of footpaths on both sides of all streets (Part 3, T7)).

Support for urban greening was mainly partial/weak because jurisdictions either only set out standards for the provision of trees or for the allocation of private open space to gardens, or addressed both of these but did not specify a percentage target for tree canopy cover. Only two jurisdictions provided a target for tree canopy cover. This was expressed in SA in terms of a 20% increase in urban green cover for councils in metropolitan Adelaide by 2045 (or maintaining tree canopy cover for jurisdictions that currently have more than 30% tree cover) (30-Year Plan for Greater Adelaide (2017 update), Part 3, Target 5). In VIC, it was described in terms of a minimum 30% target of potential canopy coverage within the public realm and open space (Precinct Structure Planning Guidelines 2021, Part 3, T13).

### Implementation and monitoring

Table 4 sets out the extent to which jurisdictions address implementation and monitoring considerations in their planning law. Most jurisdictions have a single source of default, comprehensive provisions for design and planning that apply jurisdiction-wide (e.g. the SA Planning and Design Code; Victorian Planning Provisions Planning Scheme). The legal support for design review was examined because the use of well-trained design review panels is regarded as supporting better implementation of planning laws and improved design of new developments [40]. The planning law in five jurisdictions addressed design review panels, mainly by allowing for the establishment of these. For instance, in NSW the Minister may constitute a design review panel for local government areas under the State Environmental Planning Policy No. 65, Part 3. Only two of these jurisdictions specified circumstances in the law where design review is required (e.g. in ACT, a proponent must consult the design review panel about a proposal for a ‘prescribed development proposal’ (Planning and Development Act 2007 (ACT), Sect. 138AL); in NSW, a council must not approve a draft Development Control Plan containing provisions for residential apartment development unless the council has referred the design quality provisions to any design review panel constituted to cover that council’s local government area (Environmental Planning and Assessment Regulations 2000 (NSW), clause 21A)). In other jurisdictions, advice from a design review panel was optional or recommended.

Responsibility for enforcement of the planning law was addressed by legislation in most jurisdictions (e.g. in TAS, where a planning scheme is in force, the planning authority must enforce the observance of that planning scheme in respect of all use or development within that area (Land Use Planning and Approvals Act 1993, Sect. 48); in VIC, the responsible authority for the administration and enforcement of a planning scheme is specified, and their duties are set out as including the enforcement of the planning scheme and implementing the objectives of the planning scheme (Planning and Environment Act 1987 (VIC), Sects. 13–14)). Performance monitoring was only addressed by the Planning Act in three jurisdictions (NSW, SA, TAS), and in all cases this was by specifying a responsible person or authority for evaluation and either setting out reporting obligations or requiring reporting obligations to be addressed in statutory instruments. SA additionally allowed a Minister to set performance targets in relation to any goal, policy or objective under a state planning policy, or any objectives, priorities or targets included in a planning agreement, that the Planning Commission must monitor (Planning, Development and Infrastructure Act 2016, Schedule 4, clause 1). The overall strength of addressing implementation and monitoring was mostly rated as partial (*n* = 5).

### Summative characteristics of jurisdictions

**Fig. 1:**
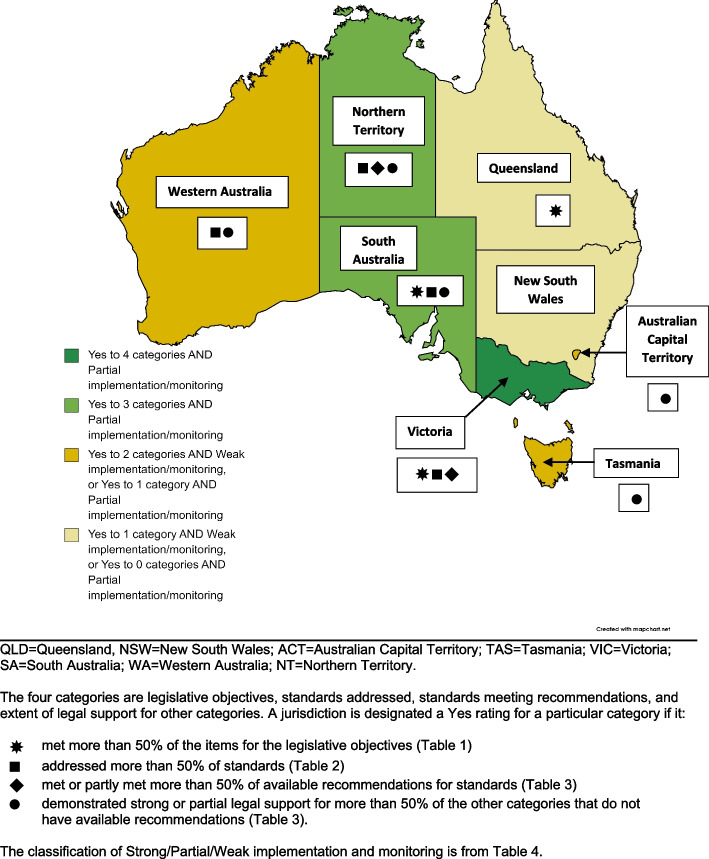
Overall summary of legal support for built environments for walking and cycling

Figure 1 presents a map summarising the extent to which there is legal support for creating built environments for walking and cycling in the eight state and territory jurisdictions, drawing upon the detailed findings shown in Tables 1, 2, 3 and 4.

## Discussion

PA is a systems issue, requiring action across multiple sectors. The WHO has identified inadequate laws and regulatory frameworks as a major barrier connected with and compounded by insufficient investment and fragmented partnerships [16], which needs to be addressed to achieve the global commitment to a 15% reduction in physical inactivity by 2030. This study reports on a comprehensive assessment of the laws that influence the built environment for PA (with a focus on walking and to some extent, cycling) in each of the states and territories of Australia. It provides insight into the coverage and adequacy of these laws, the specificity of their requirements, alignment to evidence-based recommendations, and their support for implementation and monitoring.

While PA policy research has a long history, with well-developed analytical methods and tools [41, 42], analysis of laws for PA is a nascent field [17]. Understanding the legal environment is an important component of understanding the existing system for PA, why it may not be generating the desired outcomes for PA, and potential opportunities for improvement. In order to achieve maximum system impact, the ‘rules’ of the system (which includes laws), must be addressed as one of the levers for systems change [43]. Legislative objectives provide a powerful signal about what issues should be prioritised and considered by decision makers, thus playing a role in shifting the ‘goals’ of a system, a key leverage point for systems impact [36]. While the enabling legislation does not usually set out detailed specifications, they generally enable the creation of regulations and other statutory instruments to operationalise and support the achievement of the legislative objectives [22]. Legislative objectives therefore play an important role for supporting the alignment, integration and coherence of policy across different sectors and levels of government, and stronger partnerships for systems efforts [44].

Our findings reveal disparity across Australian state and territory jurisdictions, with varying levels of legal support for promoting walking and cycling. With a few exceptions, jurisdictions showed very limited coverage of PA-promoting objectives, suggesting a lack of integrated thinking or prioritisation of this. More jurisdictions addressed broader PA-relevant objectives in their Planning Act than under their Transport Act, with health being the most common one. This may suggest greater awareness and recognition within the planning sector about the importance and relevance of health, and possibly the strength of public health advocacy in this area, compared to the transport sector. It may also demonstrate the possible influence of ‘early adopter’ jurisdictions that may be seen as inspiring other jurisdictions to follow. ACT for example, which is reforming its planning system, has put forward a Planning Bill [45] that would broaden the object of their Planning Act to address health, liveability and integrated land use and transport, aspects that are not currently covered by their existing Act [46]. The fact that the proposed wording mirrors some of the language that already exists in the SA and QLD Planning Acts, and the accompanying consultation materials specifically acknowledge that inspiration was drawn from the QLD Planning Act [46], lends support for this point. It also reinforces the potential value of scientific legal mapping, in enabling the creation of an accessible repository of systematically collected features of laws linked to relevant citations [47], that could make it easier for jurisdictions to locate and consider innovative examples of where law is being used by other jurisdictions to promote PA. This is one way of targeting ‘information flows’, one of the significant leverage points for intervening to create systems change in public health, which has been defined as the ‘movement of vital information to shift power dynamics that opens decision-making processes to more (and the right) people’ [43].

Two out of the three jurisdictions which addressed the majority of high-level factors for walking and cycling in their legislation (VIC and SA) were also the jurisdictions which addressed the most standards and demonstrated strong legal support for the greatest number of other categories. This suggests a possible relationship between greater prioritisation of PA-promoting objectives at the legislative level, and a greater commitment to achieving those objectives through the provision of specific standards. It has been suggested that being more specific and mandating health-promoting actions and procedures could assist in the achievement of related legislative objectives, as has been demonstrated in tobacco control [44]. A recent global study has shown that Australian cities are performing poorly compared to other cities throughout the world, both in terms of policies to create healthy and sustainable cities and outcomes on-the-ground in neighbourhoods [48, 49]. This suggests that policy frameworks must be strengthened through measurable standards and targets that are embedded in law, and effective mechanisms to monitor their implementation. Doing so is consistent with the WHO’s call to harness legal tools to accelerate progress on physical inactivity [16], and the recent call by the UN General Assembly for countries to ‘scale up efforts’ to ensure a clean, healthy and sustainable environment as a newly recognised human right [50].

An argument that is frequently raised against more prescriptive measures is the need to provide flexibility and discretion for innovation [4]. However, even those jurisdictions which purport to be performance-based (i.e. emphasising discretion in how planning objectives can be achieved) still recognise there is a role for prescriptive provisions (including mandatory ones), where this is required to achieve consistent and predictable results and avoid unacceptable outcomes [51].

Flexibility and certainty are by no means binary approaches. For example, in performance-based systems such as in VIC and SA, measurable criteria (which offer certainty) are sometimes provided alongside qualitative criteria (which provide flexibility), as examples of ways in which the qualitative criteria could be met to achieve the required objectives [52, 53]. They may be made mandatory where there is a sound strategic basis for that, for example, to avoid the risk of adverse outcomes where there is likely to be constant pressure for development inconsistent with planning policy [51]. Where measurable criteria are evidence-based, they can promote the widespread achievement of intended outcomes [51, 54].

There are now evidence-based criteria for numerous built environment characteristics to promote walkability [34, 35], which are likely to be generalisable to the promotion of cycling [55] although the evidence base for standards specific to cycling is less developed compared to walking. Our findings reveal a number of gaps where jurisdictions either do not provide measurable criteria addressing these characteristics in their laws, or do not fully meet the evidence-based recommendations. This calls into question the genuine commitment of governments to creating walkable, healthy, and ecologically sustainable environments. Importantly, the adoption of measurable criteria enables the implementation of laws to be monitored, such as through spatial geographic information system (GIS) measures [54]. Where laws are solely outcomes or principles-based, it is not possible to use spatial and other data to assess the strength of implementation, and progress towards supportive built environments for walking and cycling [54]. Expressing planning principles in ways that can be operationalised and tested, is also valuable for developing the evidence base about the effectiveness of laws for different outcomes. This is important to ensure that laws actually achieve their intended outcomes for PA, and if not, are refined or recalibrated accordingly [25].

The impact of adopted laws on PA outcomes is subject to whether they are implemented and enforced. Our research examined these considerations to some degree, however future research into the mechanisms and importance of implementation and enforcement of laws for PA would further identify factors that increase the beneficial impact of laws to promote PA, and generate information that could help policy makers incorporate mechanisms that have the greatest potential for success [56]. One possible approach for improving accountability and driving improvements in the planning system, may involve embedding agreed policy indicators for PA into an independent regulatory reporting framework. That framework could give an independent commissioner the functions of benchmarking laws, and monitoring and reporting on their implementation over time. Future research could use the legal data we have generated in this project, for evaluation against spatial and PA outcomes. Generating longitudinal data by updating this study over time, would support more rigorous evaluation designs [25].

A key strength of this study is that it was conducted in accordance with established and rigorous legal mapping methods [23, 57] which incorporates quality control procedures such as independent coding by two coders. It was also conducted with an interdisciplinary team with expertise in urban planning, public health, law and physical activity, thus deriving legal variables of relevance to these intersecting issues. Scientific legal mapping studies such as this one, help to address the lack of readily accessible information about the planning laws in different jurisdictions, and how they compare with each other and to evidence-based standards for promoting walking and cycling [47]. Providing accessible legal data helps to strengthen accountability within the planning system by enabling potential issues regarding the development, implementation or enforcement of relevant laws to be identified, and support evaluation and reform, as well as diffusion of innovation as jurisdictions are able to more easily learn about what others are doing [47].

However, there are some limitations with our research. As it was a cross-sectional analysis, the data only reflects the state of the law at the time of assessment. The policy context for planning is dynamic, for example, ACT is reforming its planning system [58], and WA is finalising new State Planning Policies as part of its Design WA initiative [59]. The future legal landscape can also shift unexpectedly, for example the NSW Design and Place SEPP and accompanying Urban Design Guide (excluded from the assessment as they were due to come into effect at the end of 2022 [60]), will now no longer be introduced despite being well-progressed, due to a change in the Planning Minister and approach [61]. Our assessment may be useful for providing a baseline picture of how laws currently address PA, but would need to be updated to remain current. The frequency of update and the value derived from maintaining currency would need to be considered and balanced against the resources required to complete these updates. Updating the dataset will enable changes in law to be tracked over time and support the evaluation of laws in respect of built environment and behavioural outcomes.

We also limited our analysis to state or territory-level laws; however, it is possible that local laws (or policies outside of law) address standards that were not found in the state or territory-level laws. It may be argued that local governments are better placed than state or territory governments to develop laws for certain matters due to their detailed knowledge of the local context. We chose to limit our focus to state or territory-level laws as this is consistent with calls by the UN General Assembly for countries to scale up their efforts to uphold the universal human right to a clean, healthy and sustainable environment [50], which is necessary for enabling active and sustainable lifestyles [62]. Failure to set consistent basic standards at a state or territory level will invariably lead to some local areas falling short of these expectations and exacerbating inequality [63, 64].

Our analysis was also based on selected criteria and is not exhaustive of all criteria for which there may be evidence-based recommendations for walking or cycling, for example street connectivity [34, 54]. New recommendations may also be developed for cycling as cycling-specific standards and infrastructure are implemented and evaluated around the world in line with the increasing recognition among policy makers that cycling is an important mode of sustainable transport. Any expansion of evidence-based standards relevant to walking and cycling could be captured with an update to the coding framework. Finally, our analysis only captures ‘law on the books’ (i.e. as written) and may not reflect actual practice or implementation of the law, a limitation which is generally associated with all legal mapping studies.

## Conclusion

Examination of the legal framework within and across jurisdictions is a significant and emerging area of PA policy research. Legal research can shed light on why established systems for PA may not be generating desired outcomes. Our findings reveal many opportunities for improving the goals and rules of the systems in Australian states and territories, by amending primary legislation to promote a more integrated approach to promoting active living, incorporating evidence-based standards into planning law, realigning existing standards to meet recommendations, and stronger obligations to monitor the performance of planning law. Through the creation of accessible legal data, our study contributes to improvements in information flows, enabling jurisdictions to locate relevant provisions more readily for walking and cycling, identify gaps and opportunities in their legal frameworks, and enhance cross-agency engagement between health and planning sectors.

## Supplementary Information

**Additional file 1. **

## Data Availability

The datasets generated and analysed in this study are intended to be made available from the LawAtlas.org database, or are available from the corresponding author (Tracy Nau at tracy.nau@sydney.edu.au) on reasonable request.
