# Supplementary material for: Mapping and analysis of laws influencing built environments for walking and cycling in Australia
Source: BMC Public Health. 2023 Jan 16;23:108. doi: 10.1186/s12889-022-14897-w (PMC9841659; doi:10.1186/s12889-022-14897-w)
Supplement: Supplementary file 1 — Additional file 1. [file 12889_2022_14897_MOESM1_ESM.docx]

# **Protocol for a legal assessment of laws influencing built environments for walking and cycling in Australia**

## **Date of protocol**

8 April 2022

## **Aim**

To conduct a legal assessment (a cross-sectional form of legal epidemiology) of state and territory laws that influence active built environments in Australia, thus identifying and enabling comparison of important features and variations in relevant laws.

## **Scope**

Policies with legal enforceability that address built environment considerations that are relevant to walking (and to a limited extent, cycling) in each Australian state and territory. This covered legislation (Acts, Regulations) and state/territory-wide statutory instruments. Region-level instruments covering the capital city and greater metropolitan area, were also considered. The assessment excluded non-statutory instruments (e.g. guidelines, operational policies) unless they were given legal effect such as under the planning Act or a statutory instrument. For example, the Victorian Precinct Structure Planning Guidelines are ‘guidelines’ but given legal effect under the Minister’s directions (specifically direction 12 clause 5) issued under section 12(2)(a) of the Planning and Environment Act 1987.

The review only included instruments that were in effect as of 29 November 2021. It therefore excluded any draft form instruments or instruments enacted outside of the review period. However, the latest version of any draft instruments were reviewed where available, and caution notes were made where appropriate to indicate where the response may be subject to change.

## **Project team**

Tracy Nau (TN), Sean Perry (SP), Steve Wong (SW): all trained in law; TN with additional public health experience, SP with additional planning experience, and SW a practising lawyer.

Professor Emeritus Adrian Bauman, Professor Ben Smith, Professor Emeritus Bill Bellew: physical activity and public health expertise.

Distinguished Professor Billie Giles-Corti (BGC): planning and liveability expertise.

## **Primary data collection**

### **Project dates**

Legal research was conducted between November 2020 and November 2021.

### **Dates covered in the dataset**

This is a cross-sectional dataset covering legally binding policies that were effective as of 29 November 2021.

Note that the current planning policy landscape is dynamic, with changes being considered or progressed in jurisdictions including Western Australia where a draft State Planning Policy 4.2 Activity Centres (first released in Oct 2020) and Medium Density Code (first released in Nov 2020) are both being refined and finalised, and the Australian Capital Territory which is undergoing a review and reform of its planning system.

This legal assessment may need to be updated to take account of any key changes that become effective after the review date.

### **Identification of relevant data**

An initial study was completed in April 2021^1^ which identified key legislative and statutory instruments in each state and territory that addressed built environment design considerations for walking. These were identified by reviewing the ‘statutory policies’ and ‘legislation/regulation’ listed on the National Heart Foundation’s Healthy by Design website, supplemented by web searches and review of government planning websites.

TN supplemented these searches to identify any additional documents that may be relevant by reviewing the:

- Primary Planning Act for statutory instruments enabled by the Act and inspecting those instruments for relevance and legal effect;
- List of legislative and statutory policies included by the Centre for Urban Research in its ‘Creating Liveable Cities’ (2017) report;^2^ and
- State/territory government planning websites for any other statutory instruments that may be relevant to consider.

TN consulted a subject matter expert (BGC) to review the proposed list of documents and to identify if there were any other relevant documents. We included transport, public health and climate Acts as part of our scope, consistent with an integrated systems approach to creating active built environments. [Appendix 1](#_Appendix_1._Potentially) sets out a list of laws that we included, and other documents that we considered but excluded (e.g. because they were in draft form, or were not legally enforceable).

The key areas of focus for the legal assessment were conceptualised in consultation with BGC and guided by the issues identified in recent peer-reviewed and grey literature,^3,4^ as issues of legal relevance for delivering environments that are supportive of physical activity in Australia:

1. Extent of legislative support for delivering physical activity promoting outcomes
2. Extent to which state and territory laws contained defined and measurable standards for promoting active communities
3. Alignment of standards as compared with evidence-based recommendations
4. Legislative consideration of implementation and monitoring of planning law.

TN conducted background research to investigate these areas for each jurisdiction. This informed the development of the coding scheme (see [section 6(a)](#_Development_of_the) below).

### **Data collection methods**

Once all relevant ‘laws’ were gathered, a Master Sheet was created for each jurisdiction that identified for each relevant provision of each law, the date it was last amended (if applicable) or came into force (as applicable). For laws that did not have a commencement day (only a month and year), the effective date was deemed to be the first day of the month specified.

Full text versions of the laws were obtained by searching each jurisdiction’s legislative database. Other documents were obtained by entering the name of that policy in Google, and retrieving from the relevant jurisdiction’s government planning websites. Any documents that were not publicly available were excluded from the legal assessment. The version of the full text for the relevant provisions of each law (as at 29 Nov 2021) was downloaded and extracted into Word and subsequently transferred into MonQcle^5^, a web-based software coding platform for legal epidemiology.

## **Coding**

### **Development of the coding scheme**

Using the scoping document, the project team worked together to develop the coding questions and response options. This was finalised through discussion with BGC and created in MonQcle.

### **Coding methods**

TN and SP conducted the coding. Responses were coded based on objective, measurable aspects of the law. Caution notes were recorded where an explanation of the legal text was required or deemed helpful (e.g. to provide additional clarification, note where the response could be subject to change due to proposed laws, or note that that matter was addressed under excluded documents such as non-binding policies). The coding scheme was amended as required to accommodate newly identified features in the data and completed jurisdictions were recoded accordingly. Each jurisdiction was coded using the set of coding conventions in [Appendix 2](#_Appendix_2._Coding).

## **Quality control**

### **Building and collecting the law**

SP and TN reviewed the laws identified by TN to confirm and agree on whether they met the inclusion criteria (i.e. had legal effect, were of jurisdiction-wide or region-wide application, and were relevant to the coding questions). SP and TN discussed and agreed on whether to include any other laws identified by either person as meeting the inclusion criteria.

### **Original coding**

The data was periodically exported by TN into a Microsoft Excel document to examine and address any missing entries, citations, and caution notes.

### **Redundant coding**

Each jurisdiction was coded independently by TN and SP and divergencies were resolved through consultation, and where required, by discussion with the team. The rate of divergence was checked after coding one jurisdiction and found to be 5.5%. At this point, any issues leading to confusion were addressed with clarifications to the coding scheme and conventions. The rate of divergence was checked again after coding a further two jurisdictions, with further edits made to the coding conventions. As the rate of divergence was only 2.3%, TN and SP proceeded with coding the remaining five jurisdictions. The rate of divergence for coding the remaining jurisdictions was 11.2%. All divergences were discussed between TN and SP and resolved. Whenever the coding scheme or coding conventions were amended, TN and SP revisited the dataset to ensure coding was consistent with the amended scheme and conventions.

### **Post-production statistical control**

To ensure reliability of the data, a statistical quality control procedure (SQC) was conducted at the completion of the dataset. To conduct SQC, 10% of the dataset’s parent level questions were randomly chosen using a random number generator, for each jurisdiction record. With 31 parent level questions and 8 records, this generated a total of 25 coding instances (i.e. 3-4 parent-level questions that needed to be coded for each jurisdiction). SW was brought in as a naïve coder to code this set of questions. The divergence rate was zero.

**References**

1. Nau T, Bauman A, Bellew W, Giles-Corti B, Smith BJ. An analysis of the legal framework influencing walking in Australia. Public Health Res Pract. 2022; Online early publication. <https://doi.org/10.17061/phrp32122205>
2. Arundel, J., Lowe, M., Hooper, P., Roberts, R., Rozek, J., Higgs, C., & Giles-Corti, B. (2017). Creating liveable cities in Australia. Mapping urban policy implementation and evidence-based national liveability indicators. Available from: <https://cur.org.au/project/national-liveability-report/>
3. Hooper, P., Foster, S., & Giles-Corti, B. (2019). A Case Study of a Natural Experiment Bridging the 'Research into Policy' and 'Evidence-Based Policy' Gap for Active-Living Science. *International journal of environmental research and public health, 16*(14). doi:10.3390/ijerph16142448
4. Morrison, N., Barns, S., Dunshea, A., Paine, G., Pry, J., Sajan, J., Thompson, S., Van Den Nouweland, R. (2021). Making healthy places: NSW built environment practitioners' perspectives on place-making opportunities that help deliver health and wellbeing outcomes. Marudulu Budyari Gumal. doi: <https://doi.org/10.52708/LCWA1416>
5. Temple University, Center for Public Health Law Research. MonQcle [Internet]. Available from: <https://monqcle.com>

## **Appendix 1. Included and otherwise considered laws**

The following table identifies the instruments that were included for this legal assessment, and additional documents that were considered but excluded (i.e. those marked in italics). The second column notes the source of legal enforceability (if the instrument was not an Act or Regulation), or provides a brief explanation for why the document was excluded.

| **Name of instrument** | **Source of the legal enforceability if not an Act/Regulation or reason for exclusion** |
| --- | --- |
| **VICTORIA** |  |
| Planning and Environment Act 1987 (P&E Act) |  |
| *Planning and Environment Regulations 2015* | *Not relevant to coding scheme* |
| Transport Integration Act 2010 |  |
| Climate Change Act 2017 |  |
| Public Health and Wellbeing Act 2008 |  |
| Victorian Planning Provisions (VPP) | Under the Ministerial Direction issued under s7(5) of the P&E Act, a planning scheme must include the VPPs, and responsible authorities must comply with the planning scheme (s14(c) P&E Act). |
| *Australian Standard AS 2890.3 1993 Parking facilities Part 3: Bicycle parking facilities (referenced in cl 52.34 VPP)* | *Incorporated and forms part of the VPP under cl 72.04 of the VPP. However, excluded because it was not a publicly available document.* |
| Guide to Road Design, Part 6A: Paths for walking and cycling (referenced in cl 18.02-1S VPP as a relevant policy document for promoting the use of sustainable personal transport) | Incorporated and forms part of the VPP under cl 72.04 of the VPP. Responsible authorities must comply with the planning scheme under s14(c) P&E Act. |
| Urban Design Guidelines [policy guidelines referred to in the Victoria Planning Provisions] | Under the Decision Guidelines in VPP cl 43.02-6 (Design and development overlay), the responsible authority is required to consider as appropriate the provisions of any urban design guidelines before deciding on an application, and VPP cl 65.01 requires consideration of “Any matter required to be considered in the zone, overlay or other provision” when approving an application or plan. Under the P&E Act, a responsible authority must comply with the planning scheme (s14(c)). |
| Precinct Structure Planning Guidelines 2021 | Under Ministerial Direction No. 12 (issued under s12(1A) P&E Act to manage the provision of sustainable and coordinated urban development in growth areas), a planning authority must demonstrate that the precinct structure plan or any changes to it, are in accordance with any applicable Precinct Structure Plan Guidelines. In preparing a planning scheme or amendment, a planning authority must have regard to the Minister’s directions (s12(2)(a) P&E Act). |
| *Apartment Design Guidelines for Victoria* | *Described in the VPPs as ‘policy documents’ to ‘consider as relevant’ (rather than forming part of ‘decision guidelines’) and are not an incorporated document under cl 72.04 of the VPP.* |
| *Public Transport Guidelines for Land Use and Development* | *Described in the VPPs as ‘policy documents’ to ‘consider as relevant’ (rather than forming part of ‘decision guidelines’) and are not an incorporated document under cl 72.04 of the VPP.* |
| Metropolitan Planning Strategy (i.e. Plan Melbourne 2017-2050 and addendum 2019) | Under Ministerial Direction No. 9 (issued under s12(1A) P&E Act), a planning authority must ensure that planning scheme amendments have regard to the Melbourne Planning Strategy (defined as Plan Melbourne 2017-2050). In preparing a planning scheme or amendment, a planning authority must have regard to the Minister’s directions (s12(2)(a) P&E Act). |
| *Statement of Planning Policy (e.g. Statement of Planning Policy for Macedon Ranges)* | *Only applies to specific named geographical areas declared to be distinctive areas and landscapes (i.e. meeting the criteria in s46AO P&E Act).* |
| **SOUTH AUSTRALIA** |  |
| Planning, Development and Infrastructure Act 2016 (PD&I Act) |  |
| Planning, Development and Infrastructure (General) Regulations 2017 |  |
| Passenger Transport Act 1994 |  |
| Climate Change and Greenhouse Emissions Reduction Act 2007 |  |
| Public Health Act 2011 |  |
| Planning and Design Code | Under s102(1)(a) PD&I Act 102(1)(a), the relevant authority must assess development and grant consent in respect of relevant provisions of the ‘Planning Rules’, defined to include the Planning and Design Code. |
| *Design Standards* | *None developed yet. However, if developed, they would have legal effect under s102(1)(a) PD&I Act which requires the relevant authority to assess development and grant consent in respect of relevant provisions of the Planning Rules (defined to include and the Design Standards).* |
| *State planning policies:*   - *Design quality policy* - *Integrated planning policy* - *Adaptive re-use* - *Biodiversity policy* | *Not relevant to coding scheme.* |
| State planning policy – Climate change policy | Given effect through other instruments under provisions that require the regional plan and Planning and Design Code to be consistent with any relevant state planning policy (ss64(3) and 66(3)(f) PD&I Act). However, not to be taken into account for any development assessment or decision except for impact assessed development (ss58(2), (4), 113(4), 115(5) PD&I Act). |
| Regional plans:   - 30-Year Plan for Greater Adelaide | Although this only has regional application, it was included because it covered the capital city and surrounding metropolitan area.  Regional plans are required to be considered for impact assessed development (ss113(4) and 115(5) PD&I Act). The Minister may also initiate or agree an amendment to the Planning and Design Code if consistent with a recommendation in the relevant regional plan (s75 PD&I Act). |
| *Regional Plans:*   - *Eyre and Western Region Plan* - *Far North Region Plan* - *Kangaroo Island Plan* - *Limestone Coast Region Plan* - *Mid North Region Plan* - *Murray and Mallee Region Plan* - *Yorke Peninsula Region Plan* | *Excluded because they only have regional application and do not cover the capital city.* |
| *Practice directions and practice guidelines e.g.*   - *Practice Direction 16: Urban Tree Canopy Off-set Scheme 2021 [supports operation of s197 in relation to Urban Tree Canopy Off-set Scheme]* | *Not binding, although assessment authorities will be taken to be acting consistently with the Planning Rules if they act in accordance with a practice guideline (s43(2), (5) PD&I Act). A regional plan prepared by a joint planning board must also comply with any practice direction (s64(5) PD&I Act). Nevertheless, none of the practice directions or guidelines were considered relevant to the coding scheme.* |
| *Schemes – Basic Infrastructure; General Infrastructure* | *None have been established, first piloted in 2017-2018.* |
| **WESTERN AUSTRALIA** |  |
| Planning and Development Act 2005 (P&D Act) |  |
| *Planning and Development Regulations 2009* | *Not relevant to coding scheme.* |
| *Planning and Development (Development Assessment Panels) Regulations 2011* | *Not relevant to coding scheme.* |
| Planning and Development (Local Planning Schemes) Regulations 2015:   - Model Provisions (Sch 1) – these specify that R-Codes and SPP 3.6 (Development Contributions) are to be read into local planning schemes - Deemed Provisions (Sch 2) – Part 9A (car parking) | A local planning scheme must include any model provisions prescribed by the regulations which apply to the scheme, and the regulations may also designate deemed provisions which may be enforced as part of each local planning scheme (s257A P&D Act). |
| Transport Co-ordination Act 1966 |  |
| *Metropolitan Redevelopment Authority Act 2011* | *Not relevant to coding scheme.* |
| *Metropolitan Redevelopment Authority Regulations 2011* | *Not relevant to coding scheme.* |
| *Climate Change and Greenhouse Gas Emissions Reduction Bill 2020* | *Draft legislation only; not yet passed.* |
| Public Health Act 2016 |  |
| *Region Planning Schemes:*   - *Metropolitan Region Scheme* - *Peel Region Scheme* - *Greater Bunbury Region Scheme* | *Not relevant to coding scheme.* |
| SPP7.0 Design of the built environment | Local governments must have regard to any applicable SPPs when preparing or amending a local planning scheme (s77 P&D Act), and the Minister may order an amendment to a local scheme to be consistent with a SPP (s77A P&D Act). |
| SPP7.2 Precinct Design [and accompanying Precinct Design guidelines] | Local governments must have regard to any applicable SPPs when preparing or amending a local planning scheme (s77 P&D Act), and the Minister may order an amendment to a local scheme to be consistent with a SPP (s77A P&D Act). |
| SPP7.3 (vol 1) Residential Design Code | Local governments must have regard to any applicable SPPs when preparing or amending a local planning scheme (s77 P&D Act), and the Minister may order an amendment to a local scheme to be consistent with a SPP (s77A P&D Act). In addition, the R-Codes are read as part of the local planning scheme (cl 257A and Sch 1 cl 25-26 Planning and Development (Local Planning Schemes) Regulations 2015).  It is subject to change as the draft Medium Density Code is proposed as an amendment to R-Codes Volume 1. |
| *SPP 7.3 Medium Density Residential Housing Code* | *Draft only.* |
| SPP7.3 (vol 2) Residential Design Code | Local governments must have regard to any applicable SPPs when preparing or amending a local planning scheme (s77 P&D Act), and the Minister may order an amendment to a local scheme to be consistent with a SPP (s77A P&D Act). In addition, the R-Codes are read as part of the local planning scheme (cl 257A and Sch 1 cl 25-26 Planning and Development (Local Planning Schemes) Regulations 2015).  Subject to change as the draft Medium Density Code is proposed as an amendment to R-Codes Volume 1. |
| SPP 4.2 Activity Centres for Perth and Peel (31 Aug 2010) | Local governments must have regard to any applicable SPPs when preparing or amending a local planning scheme (s77 P&D Act), and the Minister may order an amendment to a local scheme to be consistent with a SPP (s77A P&D Act). |
| *SPP 4.2 Activity Centres* | *Draft only.* |
| *Improvement Plans (define an area as shown in map) and Improvement Schemes* | *Do not have broad state or regional application but apply to very defined areas requiring special planning (see s122J P&D Act).* |
| *Redevelopment Schemes under Metropolitan Redevelopment Authority Act 2011*   - *Scarborough Redevelopment Scheme* - *Central Perth Redevelopment Scheme* - *Subiaco Redevelopment Scheme* - *Midland Redevelopment Scheme* - *Armadale Redevelopment Scheme* - *Wungong Redevelopment Scheme* | *Do not have broad state or regional application but apply to particular scheme areas that are to be revitalised and activated.* |
| *SPP 3 Urban growth and settlement* | *Not relevant to coding scheme.* |
| *Liveable Neighbourhoods [DRAFT] 2015* | *Not binding.* |
| *Liveable Neighbourhoods 2009* | *Not binding (except Element 7 below).* |
| Liveable Neighbourhoods 2009 (Element 7 – Activity centres and employment) | Cl 6.4.2(2) and 6.4.3 of SPP 4.2 requires activity centre plans to demonstrate how they satisfy Element 7 of Liveable Neighbourhoods, particularly for new urban areas. |
| Development Control Policy 1.6 Planning to support transit use and transit-oriented development | Not considered sufficiently enforceable or mandatory; cl 5.3.1(3) of SPP 4.2 only provides that activity centres should be planned in line with transit-oriented development principles which are elaborated in DCP 1.6. |
| **QUEENSLAND** |  |
| Planning Act 2016 (Act) |  |
| Planning Regulation 2017 (Sch 12A) (Regulations) | Section 43(5) of the Act provides that local planning schemes may not be inconsistent with the assessment benchmarks which cl 17 provides are those stated in schedule 11 and 12A of the Regulations. |
| *Economic Development Act 2012* | *Not relevant to coding scheme.* |
| Transport Planning and Coordination Act 1994 |  |
| *Guidelines under Part 8E of the Transport Planning and Coordination Act 1994* | *None publicly available, although a person must have regard to relevant guidelines when planning/carrying out development under the Planning Act (s8E(3) Transport Planning and Coordination Act).* |
| Transport Operations (Passenger Transport) Act 1994 |  |
| Transport Infrastructure Act 1994 |  |
| Public Health Act 2005 |  |
| State Planning Policy | The Minister (if approving the local planning scheme) is required to consider if the instrument appropriately integrates State, regional and local planning assessment policies including State Planning Policies (Ch 2 incl s18(7) Act). |
| Queensland Development Code (mandatory part) | Sections 13-14 Building Act 1975 addresses compliance with QDC. |
| *Model Code for Neighbourhood Design* | *Not binding unless adopted by a local scheme.* |
| *EDQ Guidelines*  *Priority Development Area (PDA) Guideline No. 1 Residential 30 (May 2015)* | *Not binding (unless specifically adopted by a Development Scheme); the Economic Development Act does not refer to or provide for any legal effect of these guidelines.* |
| *PDA Guideline No. 5 Neighbourhood planning and design (May 2015)* | *Not binding* |
| *PDA Guideline No. 6 Street and movement network (Feb 2019)* | *Not binding* |
| *PDA Guideline No. 8 Medium-high rise buildings* | *Not binding* |
| *PDA Guideline No. 9 Centres* | *Not binding* |
| *PDA Guideline No. 10 Industry and business areas* | *Not binding* |
| *PDA Guideline No. 11 Community facilities* | *Not binding* |
| *PDA Guideline No. 12 Park planning and design (May 2015)* | *Not binding* |
| *PDA Guideline No. 14 Environmental values and sustainable resource use* | *Not binding* |
| *PDA Practice Note No. 2 Footpath provision in residential subdivisions (March 2014)* | *Not binding* |
| *PDA Practice Note No. 6 Tree retention* | *Not binding* |
| **TASMANIA** |  |
| Land Use Planning and Approvals Act 1993 |  |
| *Land Use Planning and Approvals Regulations 2014* | *Not relevant to coding scheme.* |
| *Greater Hobart Act 2019* | *Not relevant to coding scheme.* |
| Transport Act 1981 |  |
| Passenger Transport Services Act 2011 |  |
| State Policies and Projects Act 1993 |  |
| Climate Change (State Action) Act 2008 | Note, under review as per the legislative requirement. |
| Public Health Act 1997 |  |
| *Tasmanian Planning Policies* | *Draft only.* |
| Tasmanian Planning Scheme (State Planning Provisions) | Section 9 Land Use Planning and Approvals Act 1993 establishes the Tasmanian Planning Scheme which consists of State Planning Provisions (and local provisions); s48 requires planning authorities to enforce the planning scheme in respect of all use or development in their area. |
| Regional Land Use Strategies :   - Southern Tasmania Regional Land Use Strategy^[[1]](#footnote-1)^ | Section 34(2)(e) Land Use Planning and Approvals Act 1993, provides that the Local Provisions Schedules (LPS) must (as far as practicable) be consistent with any applicable regional land use strategy. The Southern Tasmania Regional Land Use Strategy was included as it encompasses the capital city Hobart and greater metropolitan area. |
| *Regional Land Use Strategies:*   - *Living on the Coast – the Cradle Coast Regional Land Use Planning Framework* - *Northern Tasmania Regional Land Use Strategy* | *Regional application and do not cover the capital city.* |
| **ACT** |  |
| Planning and Development Act 2007 |  |
| Planning and Development Regulation 2008 |  |
| Road Transport (Public Passenger Services) Act 2001 |  |
| Climate Change and Greenhouse Gas Reduction Act 2010 |  |
| Public Health Act 1997 |  |
| Territory Plan | Section 50 Planning and Development Act 2007 prohibits the Territory, Executive, Minister or territory authority from doing anything or approving of any act that is inconsistent with the Territory Plan. |
| *Structure Plans, Concept Plans, Precinct Codes* | *While they all form part of the Territory Plan, they apply to specific geographical areas rather than the jurisdiction as a whole or types of land more broadly.* |
| *Statement of planning intent 2015* | *Not relevant to coding scheme.* |
| **NT** |  |
| Planning Act 1999 |  |
| *Planning Regulations 2000* | *Not relevant to coding scheme.* |
| Public and Environmental Health Act 2011 |  |
| NT Planning Scheme 2020 | According to s75 Planning Act 1999, development or use of land must not contravene the planning scheme, and s7 establishes a NT Planning Scheme that applies in relation to the whole of the Territory (except any land in relation to which another scheme applies or specified as being excluded). |
| Strategic framework (consisting of strategic planning policies and strategic land use plans which forms part of the NT Planning Scheme – see Part 2):   - Compact Urban Growth Policy^[[2]](#footnote-2)^ | When considering a request or proposal to amend a planning scheme, or a significant development proposal, the Minister must consider whether it is contrary to any strategic framework in the planning scheme (ss13(1)(b), 25(2)(b), 50B(3)(b) Planning Act 1999).  Note however, that the Minister may consent to a proposed development (or give a consent authority permission to approve a development) despite it being contrary to any strategic framework in the applicable planning scheme (s52(3), (4) Planning Act 1999). |
| **NSW** |  |
| Environmental Planning and Assessment Act 1979 (EPA Act) |  |
| Environmental Planning and Assessment Regulation 2000 |  |
| *Standard Instrument – Local Environmental Plan* | *Not relevant to coding scheme.* |
| Passenger Transport Act 2014 |  |
| Public Health Act 2010 |  |
| *Climate Change (Emissions Targets) Bill 2021* | *Draft legislation only.* |
| Ministerial Direction 3.4 (issued under s9.1(2) EPA Act) | Councils are required to comply with directions issued under s9.1(2) EPA Act (s9.1(3) EPA Act). |
| *Improving Transport Choice – Guidelines for planning and development* (DUAP 2001) | Direction 3.4, clause 4 requires a plan proposal to include provisions giving effect to and which are consistent with the aims, objectives and principles of this policy; s9.1(3) EPA Act requires councils to comply with Ministerial directions. |
| The Right Place for Business and Services – Planning Policy (DUAP 2001) | Direction 3.4, clause 4 requires a plan proposal to include provisions giving effect to and which are consistent with the aims, objectives and principles of this policy; s9.1(3) EPA Act requires councils to comply with Ministerial directions. |
| *Design and Place SEPP* | *Draft only, but would be given legal effect under a new direction proposed to be named the Environmental Planning and Assessment (Design Principles and Considerations) Direction 2022; s9.1(3) EPA Act requires councils to comply with Ministerial directions. However, as of 5 April 2022, the new Planning Minister decided not to introduce this.* |
| *Environmental Planning and Assessment (Design Principles and Considerations) Direction 2022 (to be issued under s9.1 EPA Act).* | *Draft only.* |
| *Urban Design Guide* | *Draft only but would be given legal effect under the Design and Place SEPP (cl 24) and new direction under s9.1 of the Act (Environmental Planning and Assessment (Design Principles and Considerations) Direction 2022). However, as of 5 April 2022, the new Planning Minister decided not to introduce this.* |
| *Greener Places Design Guide* | *Draft only. Some previous commentary suggested it would be incorporated into the Design and Place SEPP, but the draft SEPP does not refer to it. Its legal effect would need to be assessed when the Guide is finalised.* |
| State Environmental Planning Policy No. 65 (SEPP 65) – Design quality of residential apartment development | Under s3.28 EPA Act, a SEPP prevails over an LEP to extent of inconsistency. |
| Apartment Design Guide | Non-statutory instrument; however, consent authorities are required by SEPP 65 (cl 6A) to consider it in development assessment. |
| Exempt and Complying Development Code State Environmental Planning Policy (SEPP) | Under s3.28 EPA Act, a SEPP prevails over any local environmental plans, to the extent of any inconsistency. |
| Regional Strategic Plans: A Metropolis of Three Cities – the Greater Sydney Region Plan | Regional application but covers the capital city Sydney and greater metropolitan area. Section 3.8 EPA Act requires the relevant regional strategic plan to be given effect in any district strategic plan and in turn by the applicable local environmental plans (s3.8 EPA Act). |
| *Regional Strategic Plans:*   - *Central Coast* - *Central West and Orana* - *Hunter* - *Illawarra-Shoalhaven* - *New England-North West* - *North Coast* - *Riverina-Murray* - *South East and Tablelands* - *Far West* | *Regional application that does not cover the capital city and its greater metropolitan area.* |
| *District Plans: Western City, Central City, Eastern City, North, South Districts* | *Sub-regional application only.* |
| *Development Control Plans* | *Sub-regional application only developed by each local government, although there are plans to develop standard-form provisions as permitted by the EPA Act.* |
| *Integrated Transport and Land Use Guidelines* | *Not binding* |

## **Appendix 2. Coding conventions**

| **Order** | **P/C/GC/GGC^[[3]](#footnote-3)^** | **Question** | **Possible response options** | **Coding guidance** |
| --- | --- | --- | --- | --- |
|  |  | ***General coding guidance*** |  |  |
| - | - | **Where the answer is a sometimes or a maybe** | - | In situations where the answer is a sometimes, or a maybe, coders should code YES and include a caution note explaining the situation. |
| - | - | **If the collected legal text does not contain a provision to support an affirmative (YES) answer** | - | It is most likely a NO but review the original laws (saved in the shared drive) to see if any relevant laws have been overlooked. If something relevant has been missed, advise the other coder, create new legal text (for both coder’s MonQcle versions), and update the master sheet and full legal text. |
| - | - | **Answering conditional questions (i.e. where the preceding questions are coded NO / in the negative)** | - | Leave the conditional questions that follow, unanswered. |
| - | - | **Meaning of ‘standards’ (as used in the questions below)** | - | These are provisions that contain sufficient specificity to be objectively measurable (e.g. by incorporating numerical measures such as a specified distance, number of car spaces). |
|  |  | **Meaning of ‘legislation’** |  | This refers to any Act or Regulation. |
|  |  | **Meaning of ‘law’** |  | This is broader than legislation. It includes any instrument which has legal effect. If a particular matter is not addressed under law, but under an excluded document (e.g. a non-binding policy or draft law), a caution note to this effect may be made. |
|  |  | ***High-level legal support for an integrated approach to active built environments*** |  |  |
|  |  | ***Consideration of health in transport and planning*** |  |  |
| 1 | P | Is there an Act which expressly requires decision makers to consider human health as an objective, when exercising functions under the planning Act? | Yes  No | Code based on whether there is a legislative objective for health in the planning Act (or a different Act that extends this obligation to authorities exercising functions under the planning Act).  A YES response should be coded even if the requirement to consider human health as an objective only applies to the assessment of limited types of planning proposals (e.g. those likely to significantly impact transport systems). A caution note may be added to clarify. |
| 2 | P | Is liveability expressly mentioned as an objective of the planning Act? | Yes  No |  |
| 3 | P | Do any of the transport Acts expressly aim to encourage ‘public transport’? | Yes  No | Some jurisdictions have multiple transport Acts so consider all the ones that have been uploaded to MonQcle. This question is specifically focused on public transport; the next question asks about active transport. But if the Act addresses ‘active transport’ and this is defined to include public transport, then this question can be coded as YES.  The objective needs to be specific to public transport rather than ‘passenger transport’ more broadly which could encompass taxis or shared motor vehicle services, even where the objective is to encourage transport choices that are less environmentally damaging. |
| 4 | P | Do any of the transport Acts expressly aim to encourage ‘active transport’? | Yes  No | Generally, will be met if the objectives aim to promote increased active transport (or increasing walking and cycling). Where other words are used such as promoting transport choices that minimise environmental harm, consider whether there are any defined terms that reflect an objective to increase active transport, as distinct from more sustainable public transport (e.g. electric buses). |
| 5 | P | Do any of the transport Acts expressly aim to reduce private motor vehicle use? | Yes  No |  |
| 6 | P | How does the public health Act address the health impact of proposed development? | *Check all that apply*  By enabling a Minister to require an assessment or inquiry into the public health impact  By conferring a function on local councils to determine and respond to public health impact  Not addressed |  |
|  |  | ***Promotion of integrated land use and transport planning*** |  |  |
| 7 | P | Does the planning Act expressly encourage integrated land use and transport planning? | Yes  No | Note, the next question is similar but asks about transport Acts as it is sometimes dealt with in one but not the other. A YES response may be satisfied if integrated land use and transport planning is an objective of the Act, or if it is a power or function conferred on someone (e.g. an Act may enable an authority to encourage increased integration by making supporting guidelines). |
| 8 | P | Do any of the transport Acts expressly encourage integrated land use and transport planning? | Yes  No |  |
|  |  | ***Consideration of climate in planning and transport*** |  |  |
| 9 | P | Does the jurisdiction have a specific climate change Act? | Yes  No |  |
| 9.1 | C | - Does the climate legislation require decision makers to consider the potential climate impact of proposed land use and development? | Yes  No | Climate ‘legislation’ refers to any Act or Regulations (but not any non-legislative policy such as sector agreements unless the Act imposes an obligation to comply with such agreements). Merely allowing a Minister to make relevant Regulations that may address this, is insufficient unless those Regulations have been enacted and require planning authorities to consider the potential climate impact of land use and development. |
| 10 | P | Does the planning law expressly require addressing climate change? | Yes  No | This question covers ‘law’ which is broader than the Planning Act and Regulations and includes any statutory instruments with legal effect.  In ‘addressing climate change’, the planning law may require mitigation or adaptation measures to reduce greenhouse gas emissions or reduce urban heat. Requirements to improve air quality or environmental/ecological sustainability will not be considered as ‘addressing climate change’. |
| 11 | P | Do any of the transport Acts expressly aim to reduce the environmental impacts of transport? | Yes  No |  |
|  |  | ***Standards*** |  |  |
|  |  | ***Density*** |  |  |
| 12 | P | Does the planning law specify a dwelling density target for residential areas (not specifically near activity centres and/or public transit)? | Yes  No | Density ‘targets’ are different to limits; targets represent something to aim for (rather than restrict) and will usually be expressed in terms of a minimum or desirable number of dwellings per hectare. |
| 12.1 | C | - What is the dwelling density target specified for residential areas? | *Check all that apply*  Gross density  Net density  Average  <25 dph  >25 dph to <40 dph | This question addresses density targets for residential areas generally, not targets that relate to residential development located around activity centres or public transit (these are addressed by separate questions which follow).  Density targets may be expressed in terms of gross, net or average density. Select the one/s that are applicable along with the numerical target. ‘dph’ = dwellings per hectare. Dwelling density can also be expressed as a rate (e.g. 1 dwelling per 450m2). If this is not accompanied by an equivalent figure for dwellings per hectare, calculate a conversion by dividing 10,000 by the figure provided and multiplying by the number of dwellings in the rate.  If the document uses the term Net Developable Hectare (NDHA), check how this is defined in that document or by the responsible authority who developed that document. It may not necessarily be the same as Net Density. However, it can be treated as ‘Net Density’ if it includes the residential component and local roads.  If multiple targets are specified, select the most conservative measure (i.e. the lowest of the targets) and consider making a caution note to clarify. If there are multiple targets that use different measures of density (e.g. a gross target and a net target), but the density target is otherwise the same (e.g. 35dph), choose the gross measure as this will be more conservative.  If it is unclear which would be the more conservative measure (e.g. there is a target for average gross density, and net density), consider whether the planning Act addresses how to approach inconsistency in different documents (i.e. choose the measure under the document that takes precedence) and/or which of the documents are directly required to be taken into account when approving development (i.e. choose the measure under that document). |
| 12.2 | C | - What is the exact dwelling density specified for Residential? | *Insert and specify whether gross/net density* | This is a continuous variable to capture more fine-grained information about density targets. Write the exact lowest density specified. |
| 13 | P | Does the planning law specify a dwelling density target for residential areas near activity centres? | Yes – specific to activity centres  Yes – in terms of proximity to activity centres and/or public transit  No | The word ‘near’ in the question does not have a defined meaning (i.e. it does not mean a particular distance) so if the density target is expressed with reference to activity centres, that will be a YES. The target may be specific to activity centres, or together with public transit.  Activity centres may include neighbourhood centres, district centres, strategic centres. If the activity centre is classified as a particular type of centre because it meets a certain level of access to public transit, code that as ‘specific to activity centres’ rather than ‘activity centres and/or public transit’. |
| 13.1 | C | - What is the lowest dwelling density target specified for residential areas near activity centres? | *Check all that apply*  Gross density  Net density  Average  <25 dph  >25 dph to <40 dph | There may be multiple density targets (e.g. for different types of activity centres, or because the law sets a minimum as well as desirable density target). Select the lowest of these targets for this question.  See 12.1 for guidance about the term Net Developable Hectare (NDHA) and selecting the most conservative measure where there are multiple targets variously expressed in terms of gross, net or average density. |
| 13.2 | C | - What is the lowest exact dwelling density target specified for residential areas near Activity Centres? | *Insert and specify whether gross/net density and the location it applies to* | This is a continuous variable to capture more fine-grained information about density targets. Write the exact lowest density specified for activity centres. |
| 13.3 | C | - What is the highest dwelling density target specified for residential areas near Activity Centres? | *Check all that apply*  Gross density  Net density  Average  <25 dph  >25 dph to <40 dph  >70dph | There may be multiple density targets (e.g. for different types of activity centres, or because the law sets a minimum as well as desirable density target). Select the highest of these targets for this question. If there is only one density target for residential areas near activity centres, select the same response as for 13.1.  See 12.1 for guidance about the term Net Developable Hectare (NDHA) and selecting the most conservative measure where there are multiple targets variously expressed in terms of gross, net or average density. |
| 13.4 | C | - What is the highest exact dwelling density target specified for residential areas near Activity Centres? | *Insert and specify whether gross/net density and the location it applies to* | This is a continuous variable to capture more fine-grained information about density targets. Write the exact highest density specified for activity centres. |
| 14 | P | Does the planning law specify a dwelling density target for residential areas near public transit? | Yes – specific to public transit  Yes – in terms of proximity to public transit and/or activity centres  No | The word ‘near’ in the question does not have a defined meaning (i.e. it does not mean a particular distance) so if the density target is expressed with reference to public transit, that will be a YES. The target may be specific to public transit, or together with activity centres. |
| 14.1 | C | - What is the lowest dwelling density target specified for residential areas near public transit? | *Check all that apply*  Gross density  Net density  Average  <25 dph  >25 dph to <40 dph | There may be multiple density targets (e.g. because the law sets a minimum as well as desirable density target). Select the lowest of these targets for this question.  See 12.1 for guidance about the term Net Developable Hectare (NDHA) and selecting the most conservative measure where there are multiple targets variously expressed in terms of gross, net or average density. |
| 14.2 | C | - What is the lowest exact dwelling density specified for public transit? | *Insert and specify whether gross/net density* | This is a continuous variable to capture more fine-grained information about density targets. Write the exact lowest density specified for public transit. |
| 14.3 | C | - What is the highest dwelling density target specified for residential areas near public transit? | *Check all that apply*  Gross density  Net density  Average  <25 dph  >25 dph to <40 dph  >70dph | There may be multiple density targets (e.g. because the law sets a minimum as well as desirable density target). Select the highest of these targets for this question. If there is only one density target for residential areas near public transit, select the same response as for 14.1.  See 12.1 for guidance about the term Net Developable Hectare (NDHA) and selecting the most conservative measure where there are multiple targets variously expressed in terms of gross, net or average density. |
| 14.4 | C | - What is the highest exact dwelling density specified for public transit? | *Insert and specify whether gross/net density* |  |
|  |  | ***Destination accessibility*** |  |  |
|  |  | *Activity centre* |  |  |
| 15 | P | Does the planning law specify a distance within which dwellings should be located from an activity centre? | Yes  No | ‘Dwellings’ includes particular types of dwellings (e.g. retirement housing) as well as particular density types (e.g. medium density housing). An ‘activity centre’ may include references to shops.  Where the standard only applies to certain types of density development or housing, select YES. A caution note may be added to clarify. |
| 15.1 | C | - What distance is specified for the location of dwellings from an activity centre? | <800m  >800m |  |
| 15.2 | C | - What is the exact distance specified for the location of dwellings from an activity centre? | *Please write exact distance* | This is to capture more fine-grained information about distance to an activity centre. |
| 15.3 | C | Does the planning law specify a percentage target for the location of dwellings within the specified distance from an activity centre? | Yes  No |  |
| 15.3.1 | GC | - What is the percentage target specified? | < 80%  >80% |  |
| 15.3.2 | GC | - What is the exact percentage target specified? | Please write exact % |  |
| 16 | P | Does the planning law specify a walkable catchment area for activity centres? | Yes  No | This is a different question to question 15 (which relates to the proximity of dwellings from activity centres). This question is about the level of accessibility within an activity centre, which is typically expressed as a percentage or a ratio of the area within a 200m, 400m or 800m street network buffer to the area 200m, 400m or 800m Euclidian (as the crow flies) buffer around an activity centre. |
| 16.1 | C | - What is the percentage target specified for a walkable catchment for activity centres? | <60%  >60% |  |
| 16.1.1 | GC | - What types of activity centres have a walkable catchment target? | *Check all that apply*  Neighbourhood/local centres  District centres  Secondary centres  Strategic centres  Unspecified | Strategic centres include metropolitan centres. |
| 16.1.2 | GC | - What is the radius specified for the walkable catchment target for neighbourhood and local centres? | 200m  400m  800m | Complete questions 16.1.2 -16.1.6 only if they have been selected under 16.1.1. |
| 16.1.3 | GC | - What is the radius specified for the walkable catchment target for district centres? | 200m  400m  800m |  |
| 16.1.4 | GC | - What is the radius specified for the walkable catchment target for secondary centres? | 200m  400m  800m |  |
| 16.1.5 | GC | - What is the radius specified for the walkable catchment target for strategic centres? | 200m  400m  800m |  |
| 16.1.6 | GC | - What is the radius specified for the walkable catchment target for unspecified centres? | 200m  400m  800m |  |
|  |  | *Open space* |  |  |
| 17 | P | Does the planning law specify a distance within which dwellings should be located from public open space? | Yes  No | The planning law may set out relevant provisions for specific types of open space (e.g. local/neighbourhood parks, sport reserves) and/or more generally (e.g. active open space, parks and public spaces, public open space, park, quality open space).  If YES, then answer the following C question, and then for the GC questions that apply to the type/s of open space for which a distance is specified. For example, if a distance standard is set out for local/neighbourhood parks, then select that under 17.1 and complete questions 17.2-17.4.1. If there is no distance standard for local/neighbourhood parks, do not complete questions 17.2-17.4.1 (even if there are standards about the size or design of local/neighbourhood parks).  If the document expresses access to open space in terms of there being ‘adjoining’ open space, this is not the same as specifying a distance. Select NO (not a distance). A caution note may be added to clarify. |
| 17.1 | C | - What types of public open space do these standards refer to? | *Check all that apply*  Local/neighbourhood park  Active open space  Linear park/open space corridor  District park  District sport precinct  Regional/metropolitan/citywide park  Regional/metropolitan/citywide sporting precinct  Public open space/open space | Choose the term that most closely matches the term used in the document. Only select the types of open space for which there is a distance standard. |
| 17.2 | C | - What distance from a local/neighbourhood park is specified? | <400m  >400m |  |
| 17.2.1 | GC | - What is the exact distance specified from a local/neighbourhood park? | *insert* |  |
| 17.2.2 | GC | - What percentage target is specified for dwellings within the specified distance of a local/neighbourhood park? | <80  >80  None specified | If the standard applies to ‘each dwelling’ or ‘all dwellings’, this is treated as 100%. |
| 17.2.3 | GC | - What is the exact percentage specified for dwellings within the specified distance of a local/neighbourhood park? | *insert* |  |
| 17.3 | C | - Does the standard or document specify a size dimension for local/neighbourhood parks? | Yes  No | If a size dimension does not appear to be specified, check if that type of open space is defined in the document as the definition may include a size dimension. |
| 17.3.1 | GC | - What is the size dimension specified for local/neighbourhood parks? | <1.5ha  >1.5ha |  |
| 17.3.2 | GC | - What is the exact size dimension for local/neighbourhood parks? | *insert* |  |
| 17.4 | C | - Does the standard or document specify any other design criteria for local/neighbourhood parks? | Yes  No |  |
| 17.4.1 | GC | - What types of design elements are addressed for local/neighbourhood parks? | *Check all that apply*  Fit for purpose  Connectivity  Location  Diversity/adaptability  Shade  Features/facilities  Minimum area for particular types of recreational use  Shape and boundaries | Examples of each of these elements are provided below:  **Connectivity** (e.g. connected to public transport, located on bike path route, links with existing or proposed future open space)  **Location** (e.g. visible, near shops/schools/community facilities, not adjacent to main roads, consideration of topography/slopes such as location on flat land, location along foreshore/water)  **Diversity/adaptability** (e.g. diversity of uses for the space, or adaptability for different uses or community preferences)  **Shade** (e.g. natural/built shading)  **Features/facilities** (e.g. provision of amenity, playground facilities, toilets, water features, access to water supply, buffer from adjacent residential areas, buffer areas b/w active and passive open space, natural/cultural features)  **Minimum area for particular spaces** (e.g. minimum size of ovals)  **Shape and boundaries** (e.g. regular shape; % of perimeter that is road/active frontage, boundaries of x metres) |
| 17.5 | C | - What distance from active open space is specified? | <400m  >400m |  |
| 17.5.1 | GC | - What is the exact distance specified? | *insert* |  |
| 17.5.2 | GC | - What percentage target is specified for the location of dwellings within the specified distance from active open space? | <80  >80  None specified | If the standard applies to ‘each dwelling’ or ‘all dwellings’, this is treated as 100%. |
| 17.5.3 | GC | - What is the exact percentage target? | *insert* |  |
| 17.6 | C | - Does the standard or document specify a size dimension for active open space? | Yes  No | If a size dimension does not appear to be specified, check if that type of open space is defined in the document as the definition may include a size dimension. |
| 17.6.1 | GC | - What is the size dimension specified for active open space? | <1.5ha  >1.5ha |  |
| 17.6.2 | GC | - What is the exact size dimension specified for active open space? | *insert* |  |
| 17.7 | C | - Does the standard or document specify any other design criteria for active open space? | Yes  No |  |
| 17.7.1 | GC | - What types of design elements are addressed for active open space? | Fit for purpose  Connectivity  Location  Diversity/adaptability  Shade  Features/facilities  Minimum area for particular types of recreational use  Shape and boundaries | Refer to the guidance to question 17.4.1 for examples of these elements. |
| 17.8 | C | - What distance from linear parks/trails is specified? | <400m  >400m |  |
| 17.8.1 | GC | - What is the exact distance specified from linear parks/trails? | *insert* |  |
| 17.8.2 | GC | - What percentage target is specified for the location of dwellings within the specified distance from linear parks/trails? | <80  >80  None specified | If the standard applies to ‘each dwelling’ or ‘all dwellings’, this is treated as 100%. |
| 17.8.3 | GC | - What is the exact percentage target? | *insert* |  |
| 17.9 | C | - Does the standard or document specify any design criteria for linear parks/trails? | Yes  No |  |
| 17.9.1 | GC | - What types of design elements are addressed? | Fit for purpose  Connectivity  Location  Diversity/adaptability  Shade  Features/facilities  Minimum area for particular types of recreational use  Shape and boundaries | Refer to the guidance to question 17.4.1 for examples of these elements. |
| 17.10 | C | - What distance from district parks is specified? | <400m  >400m |  |
| 17.10.1 | GC | - What is the exact distance specified? | *insert* |  |
| 17.10.2 | GC | - What percentage target is specified for the location of dwellings within the specified distance from district parks? | <80  >80  None specified | If the standard applies to ‘each dwelling’ or ‘all dwellings’, this is treated as 100%. |
| 17.10.3 | GC | - What is the exact percentage target? | *insert* |  |
| 17.11 | C | - Does the standard or document specify a size dimension for district parks? | Yes  No | If a size dimension does not appear to be specified, check if that type of open space is defined in the document as the definition may include a size dimension. |
| 17.11.1 | GC | - What is the size dimension specified for district parks? | <1.5ha  >1.5ha |  |
| 17.11.2 | GC | - What is the exact size dimension specified for district parks? | *insert* |  |
| 17.12 | C | - Does the standard or document specify any design criteria for district parks? | Yes  No |  |
| 17.12.1 | GC | - What types of design elements are addressed? | Fit for purpose  Connectivity  Location  Diversity/adaptability  Shade  Features/facilities  Minimum area for particular types of recreational use  Shape and boundaries | Refer to the guidance to question 17.4.1 for examples of these elements. |
| 17.13 | C | - What distance from district sport precincts is specified? | <400m  >400m |  |
| 17.13.1 | GC | - What is the exact distance specified? | *insert* |  |
| 17.13.2 | GC | - What percentage target is specified for the location of dwellings within the specified distance from district sport precincts? | <80  >80  None specified | If the standard applies to ‘each dwelling’ or ‘all dwellings’, this is treated as 100%. |
| 17.13.3 | GC | - What is the exact percentage target? | *insert* |  |
| 17.14 | C | - Does the standard or document specify a size dimension for district sport precincts? | Yes  No | If a size dimension does not appear to be specified, check if that type of open space is defined in the document as the definition may include a size dimension. |
| 17.14.1 | GC | - What is the size dimension specified for district sport precincts? | <1.5ha  >1.5ha |  |
| 17.14.2 | GC | - What is the exact size dimension specified for district sport precincts? | *insert* |  |
| 17.15 | C | - Does the standard or document specify any design criteria for district sport precincts? | Yes  No |  |
| 17.15.1 | GC | - What types of design elements are addressed? | Fit for purpose  Connectivity  Location  Diversity/adaptability  Shade  Features/facilities  Minimum area for particular types of recreational use  Shape and boundaries | Refer to the guidance to question 17.4.1 for examples of these elements. |
| 17.16 | C | - What distance from regional/metro/citywide parks is specified? | <400m  >400m |  |
| 17.16.1 | GC | - What is the exact distance specified? | *insert* |  |
| 17.16.2 | GC | - What percentage target is specified for the location of dwellings within the specified distance from regional/metro/citywide parks? | <80  >80  None specified | If the standard applies to ‘each dwelling’ or ‘all dwellings’, this is treated as 100%. |
| 17.16.3 | GC | - What is the exact percentage target? | *insert* |  |
| 17.17 | C | - Does the standard or document specify a size dimension for regional/metro/citywide parks? | Yes  No | If a size dimension does not appear to be specified, check if that type of open space is defined in the document as the definition may include a size dimension. |
| 17.17.1 | GC | - What is the size dimension specified for regional/metro/citywide parks? | <1.5ha  >1.5ha |  |
| 17.17.2 | GC | - What is the exact size dimension specified for regional/metro/citywide parks? | *insert* |  |
| 17.18 | C | - Does the standard or document specify any design criteria for regional/metro/citywide parks? | Yes  No |  |
| 17.18.1 | GC | - What types of design elements are addressed? | Fit for purpose  Connectivity  Location  Diversity/adaptability  Shade  Features/facilities  Minimum area for particular types of recreational use  Shape and boundaries | Refer to the guidance to question 17.4.1 for examples of these elements. |
| 17.19 | C | - What distance from regional/metro/citywide sporting precincts is specified? | <400m  >400m |  |
| 17.19.1 | GC | - What is the exact distance specified? | *insert* |  |
| 17.19.2 | GC | - What percentage target is specified for the location of dwellings within the specified distance from regional/metro/citywide sporting precincts? | <80  >80  None specified | If the standard applies to ‘each dwelling’ or ‘all dwellings’, this is treated as 100%. |
| 17.19.3 | GC | - What is the exact percentage target? | *insert* |  |
| 17.20 | C | - Does the standard or document specify a size dimension for regional/metro/citywide sporting precincts? | Yes  No | If a size dimension does not appear to be specified, check if that type of open space is defined in the document as the definition may include a size dimension. |
| 17.20.1 | GC | - What is the size dimension specified for regional/metro/citywide sporting precincts? | <1.5ha  >1.5ha |  |
| 17.20.2 | GC | - What is the exact size dimension specified for regional/metro/citywide sporting precincts? | *insert* |  |
| 17.21 | C | - Does the standard or document specify any design criteria for regional/metro/citywide sporting precincts? | Yes  No |  |
| 17.21.1 | GC | - What types of design elements are addressed? | Fit for purpose  Connectivity  Location  Diversity/adaptability  Shade  Features/facilities  Minimum area for particular types of recreational use  Shape and boundaries | Refer to the guidance to question 17.4.1 for examples of these elements. |
| 17.22 | C | - What distance from public open space/open space is specified? | <400m  >400m |  |
| 17.22.1 | GC | - What is the exact distance specified? | *insert* |  |
| 17.22.2 | GC | - What percentage target is specified for the location of dwellings from public open space/open space? | <80  >80  None specified | If the standard applies to ‘each dwelling’ or ‘all dwellings’, this is treated as 100%. |
| 17.22.3 | GC | - What is the exact percentage target specified? | *insert* |  |
| 17.23 | C | - Does the standard or document specify a size dimension for public open space/open space? | Yes  No | If a size dimension does not appear to be specified, check if that type of open space is defined in the document as the definition may include a size dimension. |
| 17.23.1 | GC | - What is the size dimension specified for public open space/open space? | <1.5ha  >1.5ha |  |
| 17.23.2 | GC | - What is the exact size dimension specified for public open space/open space? | *insert* |  |
| 17.24 | C | - Does the standard or document specify any other design criteria for public open space/open space? | Yes  No |  |
| 17.24.1 | GC | - What types of design elements are addressed? | Fit for purpose  Connectivity  Location  Diversity/adaptability  Shade  Features/facilities  Minimum area for particular types of recreational use  Shape and boundaries | Refer to the guidance to question 17.4.1 for examples of these elements. |
|  |  | *Schools* |  |  |
| 18 | P | Does the planning law specify a distance within which dwellings should be located from primary schools? | Yes  No | A YES response should still be coded if the relevant provision is only for particular types of dwelling developments (e.g. high density residential). A caution note may be added to clarify.  A YES response should also be coded if the law refers to ‘schools’ without specifying or being limited to primary schools. A caution note may be added to clarify. |
| 18.1 | C | - What distance is specified? | <800m  >800m |  |
| 18.2 | C | - What is the exact distance specified? | *insert* |  |
| 18.3 | C | - Does the planning law specify a percentage target for dwellings within a specified distance from primary schools? | Yes  No |  |
| 18.3.1 | GC | - What is the percentage target specified? | <80%  >80% |  |
| 18.3.2 | GC | - What is the exact percentage target specified? | *insert* | This is a continuous variable to capture more fine-grained information. |
|  |  | ***Distance to public transport*** |  |  |
| 19 | P | Does the planning law specify a distance within which dwellings should be located from public transport? | Yes  No | A YES response should be coded even if the relevant provision is only for particular types of dwelling developments (e.g. high density residential). A caution note may be added to clarify. |
| 19.1 | C | - What does the standard specify as the distance of dwellings from public transport? | *Check all that apply*  <400m of a bus stop  >400m of a bus stop  <600m of a tram stop  >600m of a tram stop  <800m of a train stop  >800m of a train stop  <200m of public transport stop  < 400m of public transport  400 to 800m of integrated transit corridors | Note, there may be different types of standards across different documents for any one jurisdiction (e.g. one document might specify distances from a bus stop; another might specify distances from a public transport stop). |
| 19.1.1 | GC | - Does the planning law specify a percentage target for dwellings located within the specified distance from public transport? | Yes  No | A provision that applies to ‘all lots’ would be regarded as 100%. |
| 19.1.2 | GC | - What percentage target is specified? | <80%  >80% |  |
| 19.1.3 | GC | - What is the exact percentage target specified? | insert |  |
| 19.1.4 | GC | - Does the standard refer to the frequency of public transport services? | Yes  For bus only  Not at all | In order to be regarded as addressing ‘frequency’, there needs to some reference to how often the service operates. This could mean using the words ‘frequent’ or ‘regular’, or ‘every 15 mins’. |
| 19.1.5 | GC | - Where frequency is addressed, what regularity of service is specified? | Every 15 minutes  Every 30 minutes  Not specified as a time-based interval | If the regularity of service is not addressed in the provision, check whether any of the frequency-related terms used in that provision, are separately defined (e.g. ‘high frequency bus corridors’ may be defined as running every 15 minutes). An example of frequency that is not a ‘time-based interval’ is where the provision merely refers to ‘regular intervals’ without defining the length of this interval in minutes/hours. |
|  |  | ***Demand management*** |  |  |
| 20 | P | Does the planning law specify targets for the provision of car parking? | *Check all that apply*  No targets  Minimum rate  Maximum rate | If the law mostly sets out a minimum rate, but a maximum rate applies in limited circumstances, select both ‘Minimum rate’ and ‘Maximum rate’. A caution note may be added to clarify.  If the law prohibits car parking in certain areas, this would be considered as setting a maximum rate (but not where it simply maintains parking at currently approved levels). |
| 20.1 | C | - If a minimum rate is set, can parking provision be reduced below this rate? | Yes  No | In some cases, there may be discretion to provide a lesser rate if certain requirements are satisfied (e.g. considering proximity to public transport). |
| 21 | P | Does the planning law specify standards for bicycle infrastructure? | Yes  No | Standards could relate to the provision and/or the design of bike infrastructure (which includes bicycle paths as well as end-of-trip facilities). |
| 21.1 | C | What do the standards address? | *Check all that apply*  Bicycle paths  Bicycle parking  Showers  Lockers | Note the standards may address provision of these features (e.g. minimum rate of bicycle parking) and/or design of these features (e.g. width of bicycle path, design of bicycle storage); the specifics do not matter for this particular question. |
| 21.1.1 | GC | - If standards are specified for the provision of bicycle parking, which of the following developments/facilities do they apply to? | *Check all that apply*  Residential  Education  Retail  Offices  Healthcare  Sport and recreation  Public transport | Note, this question is specific to bicycle parking (the next question addresses end-of-trip facilities).  Select YES for a particular category, even if the standards do not apply to all types of developments or facilities within these categories (e.g. select the Sport and recreation option, even if there are only standards for swimming pools but not gyms).  The response options cover the main types of developments and are not intended to be exhaustive (e.g. there may be standards for other types of development such as ‘visitor accommodation’). |
| 21.1.2 | GC | - If standards are specified for the provision of other end-of-trip facilities (showers, lockers), which of the following developments/facilities do they apply to? | *Check all that apply*  Residential  Education  Retail  Offices  Healthcare  Sport and recreation  Public transport | Note, this question is specific to end-of-trip facilities.  Refer also, to the guidance to question 21.1.1. |
|  |  | ***Design*** |  |  |
|  |  | *Street block* |  |  |
| 22 | P | Does the planning law specify standards for street block size? | Yes  No | Standards may relate to the width/length for the boundary of a block, or block perimeter. They may not apply to all street blocks, but specific developments such as residential subdivision or new precincts.  Standards about the block size for a particular residence (e.g. requiring a minimum block size of 500m^2^ for a secondary residence), are not relevant to this question. |
| 22.1 | C | - What do the standards address for street block size? | *Check all that apply*  Block length  Block width  Block perimeter |  |
| 22.1.1 | GC | - If block length is specified, what is the upper limit? | <150m  >150m | The question asks for the ‘upper limit’ because the length may be expressed as a range. |
| 22.1.2 | GC | - What is the stated block length? | *insert* | If the block length is expressed as a range, write out the range (rather than only the upper limit). |
| 22.1.3 | GC | - If block width is specified, what is the upper limit? | <150m  >150m | The question asks for the ‘upper limit’ because the width may be expressed as a range. |
| 22.1.4 | GC | - What is the stated block width? | *insert* | If the block width is expressed as a range, write out the range (rather than only the upper limit). |
| 22.1.5 | GC | - If block perimeter is specified, what is the stated size? | *insert* |  |
|  |  | *Footpaths* |  |  |
| 23 | P | Does the planning law specify standards for the provision of footpaths? | Yes  No | Select YES if there are any standards for the provision of footpaths, even if they only apply to particular developments (e.g. residential subdivision). A caution note may be added to clarify this.  The provision of footpaths in or around car parking areas are not relevant to this question (as this does not relate to neighbourhood walkability). |
| 23.1 | C | - What do the standards address for the provision of footpaths? | *Check all that apply*  Width of footpath  Sides of the street where footpath is required  Increased provision near schools, shops or transit | ‘Increased provision’ may be where the law requires the provision of footpaths on more sides of the street, or a wider footpath where it connects to schools, shops or transit. |
|  |  | *Visually active frontage* |  |  |
| 24 | P | Does the planning law expressly encourage active frontage in activity centres? | Yes  No | This question is specific to activity centers which is broadly considered to be any mixed-use zones incorporating retail/commercial aspects. For the purposes of this question, ‘activity centres’ may cover city/suburban/township main street zones, urban corridor (main street) zones, local centres, urban activity centres, city/commercial zones, and similar. For the purposes of coding this question, activity centres includes shopping centres.  As guidance, refer to the Victorian [Urban Design Guidelines](https://www.urban-design-guidelines.planning.vic.gov.au/toolbox/glossary#letter_A) for a definition of ‘Activity centres’: *Activity centres within cities and towns are a focus for enterprises, services, shopping, employment and social interaction. They are where people meet, relax, work and often live. Usually well-served by public transport, they range in size and intensity of use from local neighbourhood strip shopping centres to traditional town centres and major regional centres.*  A YES will be supported if there are provisions that expressly aim to promote ‘active frontage’ in an activity centre zone (although they may use other words instead of ‘active frontage’ e.g. active street frontage, active streets, active interface, pedestrian interaction).  A YES may also be supported if the provision applies to activity centres and addresses characteristics of street frontage that are known to facilitate active frontage (even if the provision does not specifically use the words ‘active frontage’). These include the elements noted below in question 24.1 (e.g. mixed use ground floor, front facades that face and open onto the street, provision of windows and balconies that contribute to surveillance).  As guidance, refer to the Victorian [Urban Design Guidelines](https://www.urban-design-guidelines.planning.vic.gov.au/toolbox/glossary#letter_A) which defines ‘Active frontage’ as: *street frontages where there is an active visual engagement between those in the street and those on the ground and upper floors of buildings. This quality is assisted where the front facade of buildings, including the main entrance, faces and opens towards the street. Ground floors may accommodate uses such as cafes, shops or restaurants. However, for a frontage to be active, it does not necessarily need to be a retail use, nor have continuous windows. A building's upper floor windows and balconies may also contribute to the level of active frontage. Active frontages can provide informal surveillance opportunities and often improve the vitality and safety of an area. The measures of active frontage may be graded from high to low activity.* |
| 24.1 | C | - What elements are addressed to expressly encourage active frontage in activity centres? | *Check all that apply*  Coverage of windows, entrances or shopfronts  Limits to blank walls  Transparency of windows, coverings, fencing  Limits to height of fences or walls  Non-residential ground floor use (e.g. commercial, community)  Direct pedestrian access  Alfresco dining  Limits to services at street level  Limits to building setbacks  Provision of awnings/shelter  Parking away from street frontage  No specific elements | This question can be approached by looking for objectives that promote ‘active frontage’ or similar and reviewing the criteria that are set out in support of this. For the purposes of this question, the criteria specified may be qualitative or objective and measurable. The response options provided are the main ones known to promote active frontage (and are not intended to be exhaustive). |
|  |  | *Urban greening* |  |  |
| 25 | P | Does the planning law specify standards for the provision of trees? | Yes  No | A YES response should be coded if there are standards for either street trees (i.e. planted in the public domain) or trees in the private realm, and if there are standards applying to selected developments (e.g. residential subdivision). |
| 25.1 | C | - What elements do the standards address regarding the provision of trees? | *Check all that apply*  Spacing  Size of tree  Canopy cover of tree  Number of trees  Placement of trees | **Spacing** may mean one tree every 15m (typically for a linear area such as a street), or one tree per 30m^2^ of deep soil area (typically for a lateral area). It is not spacing for the purposes of this question, if the deep soil area is described in terms of the amount of deep soil that should be provided per tree.  **Size** may mean small, medium, large as defined by the document.  **Canopy cover** may refer to the indicative canopy diameter when the tree reaches maturity.  **Number of trees** may refer to a minimum number of trees per dwelling, or a rate of provision of trees in an area.  **Placement** is different to spacing. It may cover provisions that require the co-location of new trees with existing trees, and the sides of the road where trees should be planted, as well as whether the tree is planted on or off site or located in private open space or a communal courtyard. |
| 26 | P | Does the planning law specify any standards for the allocation of private open space for gardens/planting/soft landscaping? | Yes  No | Where the law sets out qualitative criteria that dwellings have ‘reasonable space’ for the planting of gardens and landscaping, this is not specific or measurable enough to be a standard. However, a YES can be justified if there is also an accompanying provision imposing limits on the % coverage that can be occupied by a dwelling or requiring minimum % of private open space. |
| 26.1 | C | - How do the standards address the allocation of private open space for gardens/planting/soft landscaping? | *Check all that apply*  Minimum dimensions for gardens/planting/soft landscaping  Minimum % of site set aside for gardens/planting/soft landscaping  Maximum % site coverage for buildings | A ‘maximum % site coverage for buildings’ can be selected even if there is no additional provision/criteria for the allocation of gardens/planting/soft landscaping. Compared to the other response options, it is a lower quality standard as it just restricts the amount of land that can be built over but does not necessarily ensure an adequate amount of space for gardens. |
| 27 | P | Does the planning law specify a percentage target for increasing tree canopy cover? | Yes  No | This can be expressed in terms of a target to increase existing canopy cover by X% or increase canopy cover to Y%. |
| **C.** |  | ***Implementation and monitoring*** |  |  |
| 28 | P | Does the planning law establish a comprehensive set of default provisions that apply to development assessment at a jurisdiction-wide level? | Yes  No | A ‘set of default provisions’ means a single source of provisions that apply as a matter of standard across the jurisdiction. ’Comprehensive’ means it incorporates a scheme of detailed planning and design provisions for the use and development of land in relation to different zones and specified issues. |
| 29 | P | Does the planning legislation (i.e. planning Act, planning Regulations) specify responsibility for the enforcement of planning law? | Yes  No | This is specific to the planning Act or Regulations. The legislation may identify who is responsible for the enforcement of the Act, or the enforcement of a planning scheme. The aim of this question is to understand whether there is a person or authority who is responsible for ensuring compliance; this may be by conferring functions to do so, or obligations (but not by conferring powers which are discretionary). Some illustrative examples are provided below as guidance.   - If the Act does not expressly refer to ‘enforcement’ but imposes an obligation on an authority to ensure compliance with the Act/scheme, that would support a YES. - If there are provisions that confer functions or obligations on an inspector/authority to monitor and enforce compliance, that would support a YES. - If the Act gives a Minister powers to order a local council to enforce compliance of a scheme (e.g. following an investigation), that would be a NO as this would be considered too indirect an obligation (the Minister has to first choose to investigate, and then decide to order the council to enforce compliance). - If the Act merely confers powers to inspect or issue an enforcement notice, that would be a NO. |
| 30 | P | How does the planning law address the establishment of design review panels? | Requires the establishment of design review panels  Allows for the establishment of design review panels  Allows for the establishment of committees (but not design review panels specifically)  Not addressed | Note that this question requires consideration of the planning ‘law’ as this matter may be addressed in statutory instruments rather than Acts/Regulations. A YES is supported even if the law only provides for the establishment of design review panels for certain local government areas or to assess certain types of development. |
| 30.1 | C | - Does the planning law specify any circumstances where advice from a design review panel would be required? | Yes  No | This question relates to whether there are mandatory circumstances where design review panel advice needs to be obtained.  Select YES if there are *any* circumstances where development proposals need to be referred to a design review panel.  If there are provisions which merely recommend design review in certain situations, this is not sufficient to satisfy a YES. |
| 31 | P | Does the planning Act address performance monitoring of planning law objectives? | Yes  No | This is limited to the planning Act. Note that a statutory instrument may address monitoring, but there would need to be a provision in the enabling Act that specifically requires the statutory instrument to address monitoring, for a YES to be satisfied.  Further guidance about whether a provision addresses monitoring, is provided below:   - The provision does not need to *require* monitoring, but it has to *address* this (e.g. it can allow the Minister to set targets for monitoring). - A NO response should be coded if the obligation is only expressed in terms of providing information/reports ‘as required’ by the Minister (this is not regarded as specific enough).   Further guidance about whether the monitoring obligation addresses planning law objectives, is provided below:   - A YES is supported if there is a legislative obligation to review the implementation of a statutory instrument where that statutory instrument is expressly required to further the objectives of the Act, or if the instrument specifically identifies the objectives of the Act that it seeks to further. - A NO response should be coded, if the monitoring provisions are not in relation to the planning law objectives, but other matters (e.g. effectiveness of an offset policy, environmental impact). - A NO response should be coded if the obligation is just to review the Act or keep particular instruments under review, without further details about what they should be reviewing them for. |
| 31.1 | C | How does the planning Act address performance monitoring of planning law objectives? | *Check all that apply*  Allows a Minister to set targets Allows a Minister to appoint a responsible authority for evaluation  Specifies a responsible person or authority for evaluation  Requires reporting obligations to be addressed in statutory instruments  Sets out obligations for reporting |  |

1. Covers Southern Tasmania and metro Hobart. Most populous urban area in Tas (p1 of Strategy). [↑](#footnote-ref-1)
2. Applies to higher density residential proposals in urban brownfield and greenfield localities in NT. An application or Area Plan must demonstrate compliance with all the KPIs in s4 of the policy or else demonstrate an alternative solution that achieves the objectives of the policy (s3.2 Compact Urban Growth Policy). [↑](#footnote-ref-2)
3. P=parent question; C=child question (conditional on the parent); GC=grandchild question (conditional on the child); GGC=great grandchild (conditional on GC). [↑](#footnote-ref-3)
